# Green Synthesis of Diphenyl‐Substituted Alcohols *Via* Radical Coupling of Aromatic Alcohols Under Transition‐Metal‐Free Conditions

**DOI:** 10.1002/open.202400139

**Published:** 2024-08-22

**Authors:** Ha V. Le, Vy T. B. Nguyen, Huy X. Le, Tung T. Nguyen, Khoa D. Nguyen, Phuoc H. Ho, Thuong T. H. Nguyen

**Affiliations:** ^1^ Faculty of Chemical Engineering Ho Chi Minh City University of Technology (HCMUT) 268 Ly Thuong Kiet 740010 District 10, Ho Chi Minh City Vietnam; ^2^ Vietnam National University Ho Chi Minh City Linh Trung Ward 720400 Thu Duc City, Ho Chi Minh City Vietnam; ^3^ Chemical Engineering, Competence Centre for Catalysis Chalmers University of Technology Gothenburg SE-412 96 Sweden

**Keywords:** 1,3-diphenylpropan-1-ol, aromatic alcohol, radical coupling, transition-metal-free condition, green synthesis

## Abstract

Alcohols are common alkylating agents and starting materials alternative to harmful alkyl halides. In this study, a simple, benign and efficient pathway was developed to synthesize 1,3‐diphenylpropan‐1‐ols *via* the *β*‐alkylation of 1‐phenylethanol with benzyl alcohols. Unlike conventional borrowing hydrogen processes in which alcohols were activated by transition‐metal catalyzed dehydrogenation, in this work, *t‐*BuONa was suggested to be a dual‐role reagent, namely, both base and radical initiator, for the radical coupling of aromatic alcohols. The cross‐coupling reaction readily proceeded under transition metal‐free conditions and an inert atmosphere, affording 1,3‐diphenylpropan‐1‐ol with an excellent yield. A good functional group tolerance in benzyl alcohols was observed, leading to the production of various phenyl‐substituted propan‐1‐ol derivatives in moderate‐to‐good yields. The mechanistic studies proposed that the reaction could involve the formation of reactive radical anions by base‐mediated deprotonation and single electron transfer.

## Graphical abstract

Insert text for Table of Contents here. A series of 1,3‐diphenylpropan‐1‐ol derivatives were successfully obtained *via* the *t‐*BuONa‐mediated radical coupling of stable and available substrates involving 1‐phenylethanol and benzyl alcohols without the need of any external oxidants and transition metal catalysts. The experimental results demonstrated the crucial role of a strong base in the steps of the conversion to the desired product. From the viewpoint of green chemistry, the applied‐here pathway is simple, less toxic, step‐ and cost‐efficient.

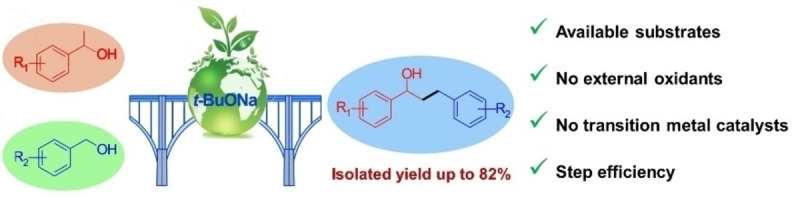

## Introduction

Carbon‐carbon (C−C) bond formation is one of the fundamental reactions in organic synthesis.[^1^, ^2^, ^3^] The design and development of methodologies for the C−C bond formation have attracted much attention in both academic and industrial research.[^4^, ^5^] Traditionally, the C−C bond formation, especially the C‐alkylation of carbonyl compounds has been achieved by nucleophilic substitution reactions with alkyl halides or other activated derivatives.[^1^, ^6^, ^7^, ^8^] However, toxic, expensive, and highly reactive reagents are required for this classical pathway, resulting in the generation of large stoichiometric amounts of hazardous wastes.[^9^, ^10^, ^11^] Therefore, an alternative to such reagents for this type of reaction is highly desirable. Alcohols are promising surrogates for these toxic reagents due to the possibility of obtaining them from abundantly available and underutilized biomass (lignocellulose).[^12^, ^13^, ^14^, ^15^] Recently, borrowing hydrogen or hydrogen auto‐transfer has shown their importance in building C−C bonds from alcohols as green and sustainable syntheses. These methods are featured by the alcohol catalytic dehydrogenation towards carbonyl compounds, followed by a condensation reaction between a suitable nucleophile and subsequent hydrogenation by the “borrowed” hydrogen.^[16]^ Overall, such approaches overcome the inherent limitations of traditional methods and produce desired products with high atom efficiency and water as the only byproduct.

**Figure 1:**
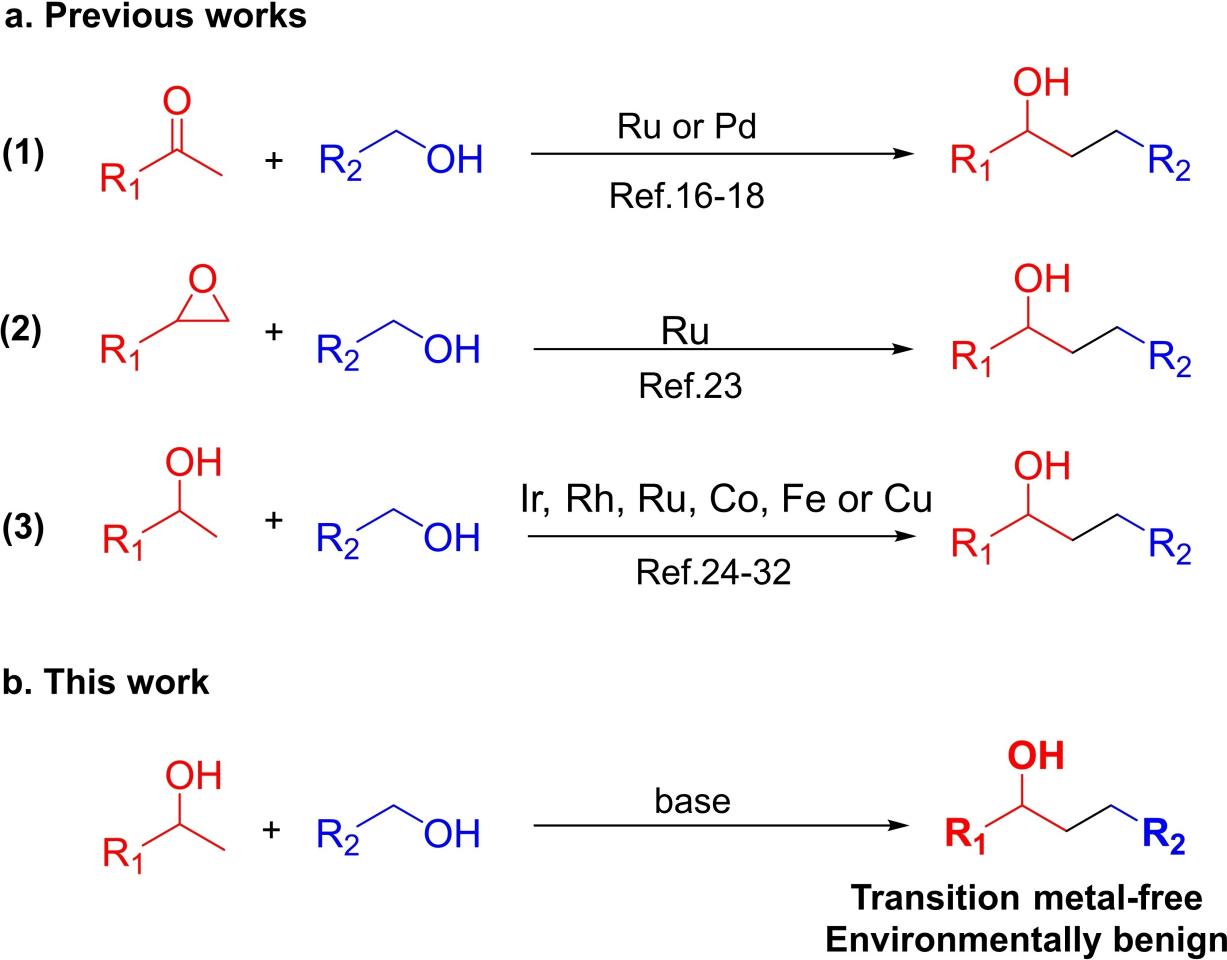
Synthesis of 1,3‐diphenylpropan‐1‐ol in (a) previous works and (b) this work.

With these advantages, many efforts to apply the borrowing hydrogen or hydrogen auto‐transfer model have been reported over the last decade (Figure 1a). A high atom efficiency was obtained in the *α*‐alkylation of methyl ketone derivatives with primary alcohols through a hydrogen auto‐transfer process in the presence of Ru or Pd complex catalysts **(1)**.[^17^, ^18^, ^19^] Nevertheless, the use of excess primary alcohol and methyl ketones as starting materials forced the reaction to yield the corresponding secondary alkylated alcohols *via* Meerwein–Ponndorf–Verley/Oppenauer redox processes.[^17^, ^20^, ^21^, ^22^, ^23^] Furthermore, Maji and co‐workers investigated the ruthenium‐catalyzed synthesis of *β*‐alkylated secondary alcohols *via* the regioselective ring‐opening of epoxides with primary alcohols **(2)**.^[24]^ The approach was indeed highly efficient but limited by drawbacks such as using hazardous solvents and a large amount of oxidants for epoxide preparation. In recent years, *β*‐alkylation of secondary alcohols with primary alcohols has been widely studied by using transition‐metal catalysts (Ru, Ir, Rh, Co, Fe, or Cu catalysts, …) **(3)**.[^25^, ^26^, ^27^, ^28^, ^29^, ^30^, ^31^, ^32^, ^33^] Obviously, these protocols allowed the *β*‐alkylation *via* a borrowing hydrogen strategy from more stable and available substrates. However, these methodologies still suffer from the utilization of an equimolecular amount of base, long reaction time, or low selectivity of the products. Moreover, noble metal complexes or external ligands used for catalyst activation are not only toxic, and expensive, but also more prone to metal contamination into products, leading to limited pharmaceutical, biochemical, and industrial applications of target products.[^34^, ^35^, ^36^] Herein, the direct synthesis of 1,3‐diphenylpropan‐1‐ols from primary alcohols as potential alkylating agents with secondary alcohols under transition metal‐free conditions was investigated (Figure 1b). Interestingly, 1,3‐diphenylpropan‐1‐ols are known to be potential synthetic precursors of flavans that possess many pharmacological activities such as anticarcinogenic, anti‐inflammatory, antioxidant, and antimalaria.[^37^, ^38^, ^39^] Therefore, the simple and efficient pathway in this study has the potential to be applied to the synthesis of flavan precursors.

## Experiment

### Materials

All chemicals were purchased from Merck, Sigma‐Aldrich, Acros, Energy Chemical, Chemsol, and Xilong companies. These chemicals were used as received without any further purification unless otherwise noted.

### Characterization of Organic Compounds

Gas chromatographic (GC) analyses were performed on a Shimadzu GC 2010‐ Plus equipped with a flame ionization detector (FID) and an SPB‐5 column (length=30 m, inner diameter=0.25 mm, and film thickness=0.25 μm). The oven of the GC was initially held at 100 °C for 1 min and heated to 280 °C with a ramp rate of 40 °C min^−1^ and subsequently held at 280 °C for another 4.5 min before being cooled down to 100 °C. The inlet and detector temperatures were kept constant at 280 °C.

Mass spectra (MS) were recorded on a Shimadzu GC‐MS‐QP2010 Ultra with a ZB‐5MS column (length=30 m, inner diameter=0.25 mm, and film thickness=0.25 μm). The sample was held at 50 °C for 2 min before being heated to 280 °C at 10 °C min^−1^ and finally held at 280 °C for 10 min. The inlet temperature was constantly set at 280 °C. The mass spectra were compared with references from the NIST library.

Nuclear magnetic resonance spectra (^1^H‐NMR and ^13^C‐NMR) were recorded in DMSO‐*d_6_* using residual solvent peak or tetramethylsilane as a reference on a Bruker AV 500 spectrometer.

Analytical thin layer chromatography (TLC) plates were purchased from Merck KGaA (silica gel 60 F254). Visualization of the chromatogram was performed by ultraviolet light (254 nm or 365 nm). Column chromatography was carried out using silica gel (230–400 mesh).

### Synthesis of 1,3‐diphenylpropan‐1‐ol

In a typical experiment for the synthesis of 1,3‐diphenylpropan‐1‐ol, 1‐phenylethanol (91.6 mg, 0.75 mmol), benzyl alcohol (54.1 mg, 0.5 mmol), *t‐*BuONa (9.6 mg, 0.1 mmol) and toluene (0.75 mL) were added to a 12‐mL pressurized vial. The mixture was purged with argon in 90 s before being tightly sealed to ensure an inert atmosphere. The reaction was performed at 140 °C for 20 h under vigorous stirring. Upon the completion, the reaction mixture was cooled to room temperature and a predetermined amount of diphenyl ether was added to the reaction mixture as an internal standard. Subsequently, an aliquot of the resulting mixture was withdrawn and quenched with brine (2.0 mL). The organic phase was extracted into ethyl acetate (3.0 mL), dried over anhydrous Na_2_SO_4_, filtered by a cotton layer, and analyzed by gas chromatography to determine the yield of the desired product.

For the isolation of 1,3‐diphenylpropan‐1‐ol, the product was purified by column chromatography, using silica gel as a stationary phase and an ethyl acetate/hexane mixture (1/5 vol., R_f_=0.30) as an eluent, affording 1,3‐diphenylpropan‐1‐ol as a colorless oily liquid. The product structure was further confirmed by GC‐MS, ^1^H‐NMR, and ^13^C‐NMR.

## Results and Discussion

**Figure 2:**
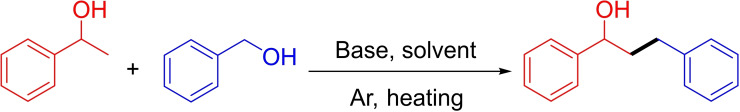
Base‐mediated coupling of 1‐phenylethanol with benzyl alcohol.

**Figure 3:**
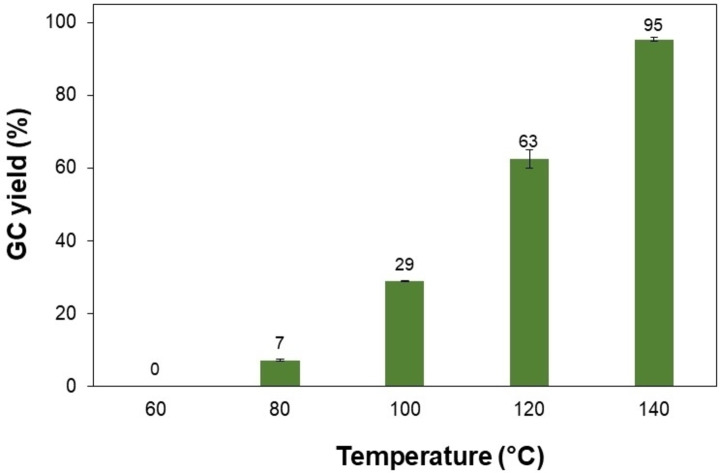
Effect of temperature on the 1,3‐diphenylpropan‐1‐ol yield. Reaction conditions: 1‐phenylethanol (0.75 mmol); benzyl alcohol (0.5 mmol); *t*‐BuONa (0.2 equiv.); toluene (0.75 mL); argon atmosphere, 20 h.

*β*‐Alkylation of 1‐phenylethanol with benzyl alcohol to yield 1,3‐diphenylpropan‐1‐ol in the presence of a base was selected as the model reaction (Figure 2) for screening the reaction conditions to improve the product yield. Initially, the influence of the reaction temperature on the C2‐benzylation of 1‐phenylethanol was explored (Figure 3). The cross‐coupling reaction was carried out in the temperature range from 60 to 140 °C for 20 h in the presence of 0.2 equiv. of *t*‐BuONa as a base and 0.75 mL of toluene as a solvent under an argon atmosphere. Elevating the reaction temperature was found to be necessary for this transformation since only a trace amount of the target product was detected at below 80 °C. Conducting the reactions at a higher temperature would lead to better performances. The reaction carried out at 100 and 120 °C gave significantly higher product yields of 29 % and 63 %, respectively. The best performance was observed when conducting the reaction at 140 °C with a yield of 95 %. Indeed, the base‐mediated alkylation of 1‐phenylethanol with benzyl alcohol was performed in a temperature range of 130–150 °C in the earlier studies, implying that this temperature range was appropriate for the selective activation and coupling of C(sp^3^)−H and C(sp^3^)−H bonds.[^40^, ^41^, ^42^, ^43^, ^44^, ^45^]

**Figure 4:**
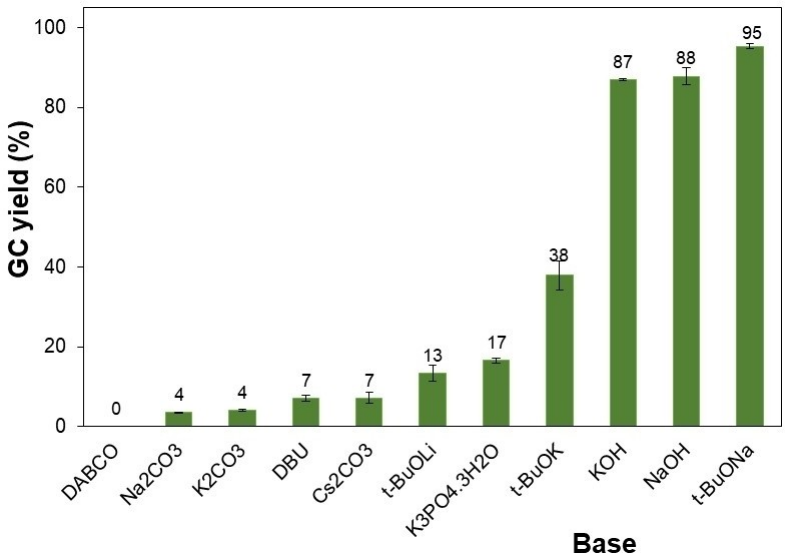
Effect of different bases on the 1,3‐diphenylpropan‐1‐ol yield. Reaction conditions: 1‐phenylethanol (0.75 mmol); benzyl alcohol (0.5 mmol); base (0.2 equiv.); toluene (0.75 ml); 140 °C; argon atmosphere, 20 h. (DABCO: 1,4‐diazabicyclo[2.2.2]octane; DBU: 1,8‐diazabicyclo[5.4.0]undec‐7‐ene).

**Figure 5:**
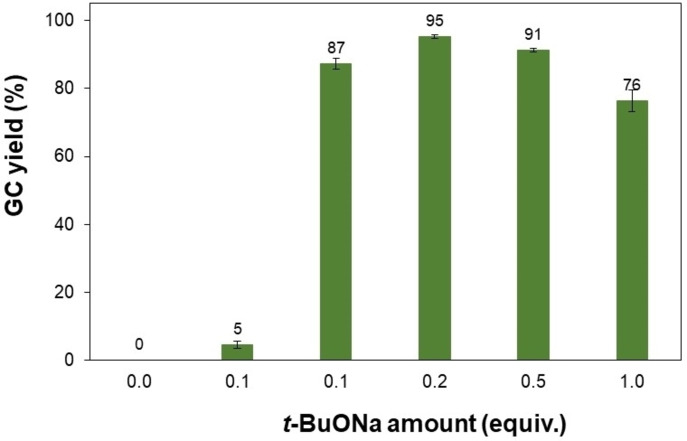
Effect of the t‐BuONa amount on the product yield. Reaction conditions: 1‐phenylethanol (0.75 mmol); benzyl alcohol (0.5 mmol); toluene (0.75 ml); 140 °C; argon atmosphere; 20 h.

According to a mechanism proposed by Kobayashi *et al*. in 2021 for the iridium nanoparticle‐catalyzed synthesis of 1,3‐diphenylpropan‐1‐ol from benzyl alcohol and 1‐phenylethanol, a base plays an important role in the steps including the oxidation of alcohols, aldol condensation, and final reduction to give *β*‐alkylated product.^[46]^ Therefore, various organic and inorganic bases were examined for this metal catalyst‐free reaction (Figure 4). It was observed that this transformation generally depended on the base strength. Inorganic salt‐based bases including Na_2_CO_3_, K_2_CO_3_, CsCO_3_, or K_3_PO_4_ were found to be incompatible with this reaction with negligible or low yields of 4–17 %. Poor results were also obtained for the reactions using organic bases (DABCO, DBU). These bases were not strong enough for the alkylation of these two alcohols.[^32^, ^45^, ^47^] As can be expected, the reactions using alkali hydroxides such as KOH and NaOH gave high yields of approximately 88 %. Interestingly, the application of alkali *tert‐*butoxides led to various results, suggesting that the alkali counter ion had a significant impact on the base performances. In particular, *t*‐BuOLi was inefficient, affording a low yield of 13 % while the *β*‐alkylation of 1‐phenylethanol proceeded more readily in the presence of *t*‐BuOK, producing 1,3‐diphenylpropan‐1‐ol in a 38 % yield. The best yield of 95 % was obtained when *t*‐BuONa was used for the reaction under identical conditions. Next, the reaction of 1‐phenylethanol and benzyl alcohol was investigated by varying the *t*‐BuONa amount. No *β*‐alkylated product was observed in the absence of *t*‐BuONa (Figure 5), implying the irreplaceable role of base for this coupling reaction. Obviously, the formation of 1,3‐diphenylpropan‐1‐ol could be considerably accelerated to the 95 % yield by increasing the base amount to 0.2 equiv. However, losses in the product yield were observed when more than 0.2 equiv. of *t*‐BuONa was used probably due to base‐promoted side reactions of generated carbonyl intermediates hindering the interaction of substrates.

**Figure 6:**
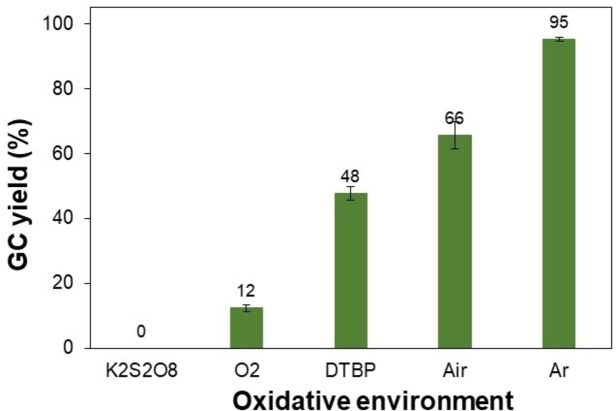
Effect of the oxidative environment on the product yield. Reaction conditions: 1‐phenylethanol (0.75 mmol); benzyl alcohol (0.5 mmol); *t‐*BuONa (0.2 equivalents); toluene (0.75 mL); liquid oxidant (if any, 4 equiv.), 140 °C, 20 h. (DTBP: di‐*tert*‐butyl peroxide).

In addition, different oxidative environments were tested for the reaction (Figure 6). The results showed that the use of oxidizing agents for generating free radicals at high temperatures was unsuitable for this reaction. Indeed, there is no product detected after 20 h in the presence of K_2_S_2_O_8_ as the oxidation while the reaction could proceed slowly for the case of DTBP, affording a poor yield of 48 %. It was hypothesized that both K_2_S_2_O_8_ and DTBP can partly generate *α‐*hydroxybenzyl radicals from benzyl alcohol, which is unreactive with intermediate acetophenone.^[48]^ Notably, a decrease in the 1,3‐diphenylpropan‐1‐ol yield was observed as the reaction was carried out under air or oxygen. This result was consistent with the previous study reported by El‐Sepelgy and co‐workers for manganese‐catalyzed reaction under an inert argon atmosphere with a high isolated yield of 92 % for the same reaction.^[41]^ In particular, the inert argon atmosphere emerges as the best choice, producing 1,3‐diphenylpropan‐1‐ol in the 95 % yield.

**Figure 7:**
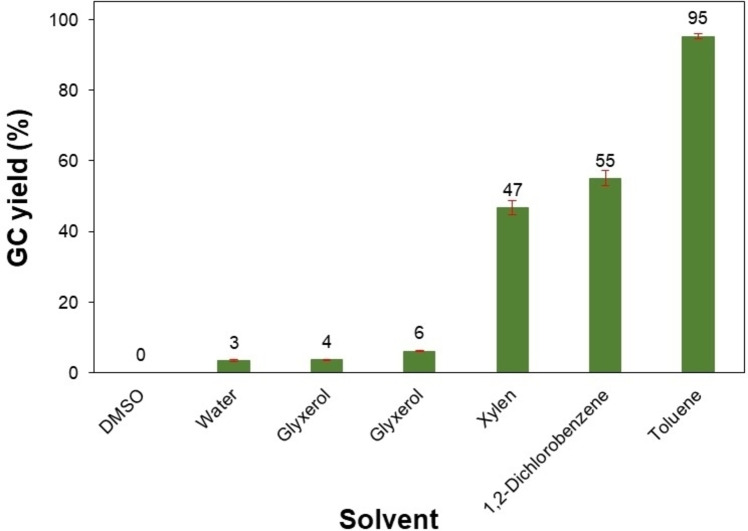
Effect of different solvents on the product yield. Reaction conditions: 1‐phenylethanol (0.75 mmol); benzyl alcohol (0.5 mmol); *t‐*BuONa (0.2 equivalents); solvent (0.75 mL); 140 °C; argon atmosphere; 20 h. (DMSO: dimethyl sulfoxide).

**Figure 8:**
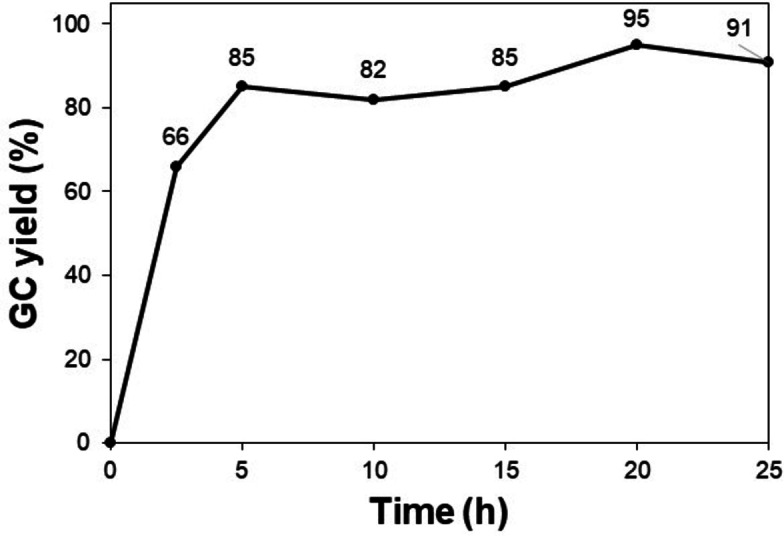
Effect of the reaction time on the product yield. Reaction conditions: 1‐phenylethanol (0.75 mmol); benzyl alcohol (0.5 mmol); *t*‐BuONa (0.2 equivalents); toluene (0.75 mL); 140 °C; argon atmosphere.

Under identical conditions, the effect of various solvents including DMSO, water, glycerol, DMF, xylene, 1,2‐dichlorobenzene, and toluene on the formation of the desired product was examined (Figure 7). It was found that this transformation was highly dependent on the solvent nature. Significant decreases in the product yield were observed when the reaction was performed in either protic or aprotic polar solvents such as DMSO, water, glycerol, and DMF, which might join the proton exchange and inhibit the performance of *t‐*BuONa for the main transformation. Interestingly, non‐polar solvents including xylene or 1,2‐dichlorobenzene could promote the formation of 1,3‐diphenylpropan‐1‐ol with a yield of approximately 50 %. Notably, toluene was found to be the most efficient solvent and gave the desired product in an excellent yield of 95 %. Furthermore, the great efficiency of this pathway was demonstrated *via* the kinetics study (Figure 8). 1,3‐Diphenylpropan‐1‐ol was obtained in a high yield of 85 % within the first 5 h of the reaction course. Further prolonging the reaction time to 20 h resulted in a minor yield improvement by 10 %.

**Figure 9:**
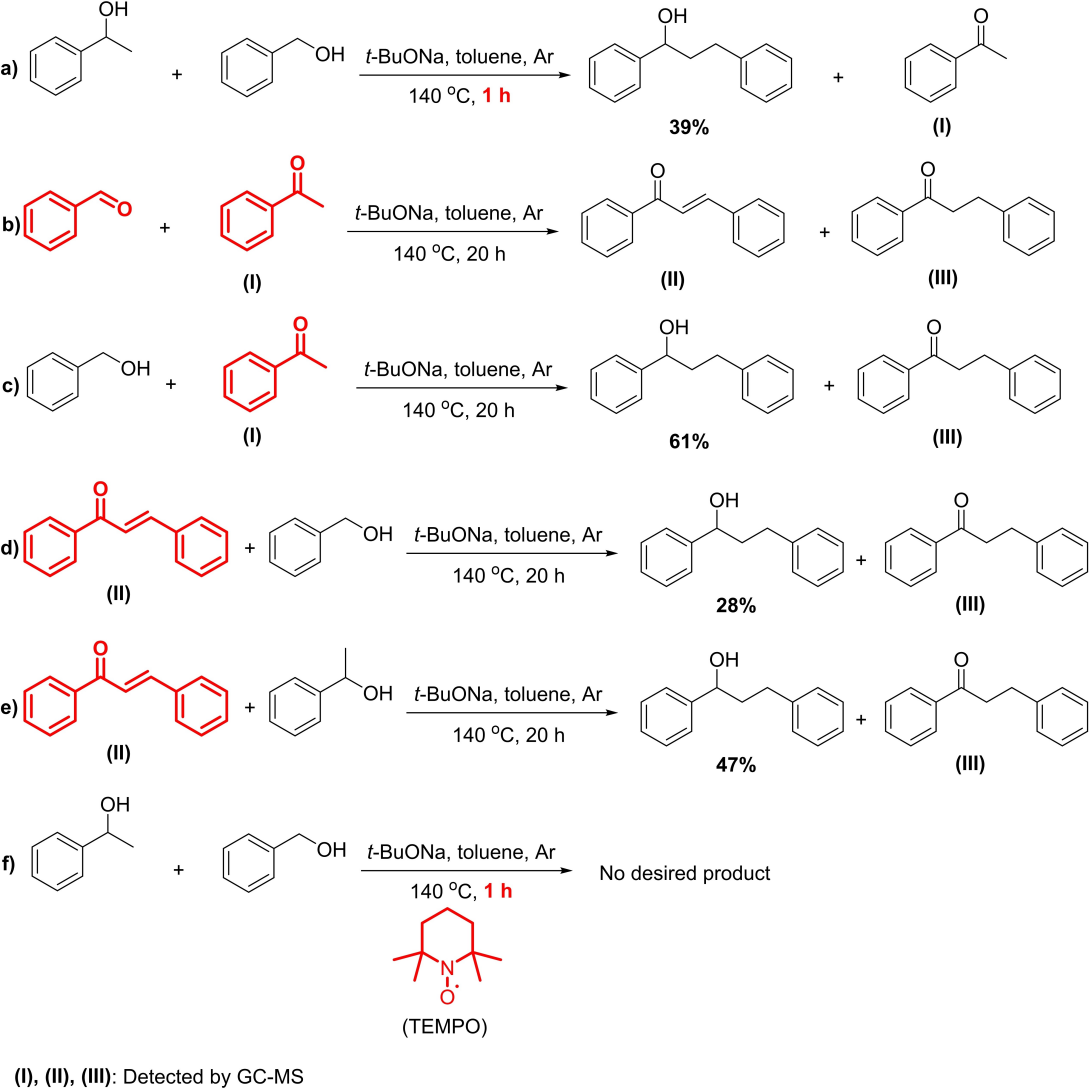
Control experiments.

To investigate the reaction mechanism, various control experiments were performed (Figure 9). Acetophenone **I** was detected as an intermediate while no benzaldehyde could be detected due to the anaerobic conditions when the reaction was monitored by GC/MS (Figure 9a). It was previously reported the base‐mediated oxidation of benzyl alcohol to benzaldehyde required an atmosphere of dioxygen or air.[^49^, ^50^, ^51^] Therefore, the coupling of aromatic alcohols in the presence of *t‐*BuONa under an inert atmosphere was unlikely to occur in the Meerwein–Ponndorf–Verley/Oppenauer redox pathway.[^52^, ^53^] On the other hand, the reaction of acetophenone with benzaldehyde produced a mixture of intermediate ketones including 1,3‐diphenylpropen‐1‐one **II** and 1,3‐diphenylpropan‐1‐one **III** (Figure 9b). However, 1,3‐diphenylprop‐2‐en‐1‐ol was not observed under these alcohol‐free conditions, indicating the essential role of starting alcohols as hydrogen sources for the intermediates. As can be expected, the reaction of acetophenone with benzyl alcohol under identical conditions gave 1,3‐diphenylpropan‐1‐ol a yield of 61 % along with a large amount of **III** (Figure 9c). Furthermore, the treatment of **II** in the presence of benzyl alcohol or 1‐phenylethanol under the basic conditions also led to the formation of the desired product and a minor amount of **III** (Figure 9d **&e**). It should be noted that no traces of the unsaturated alcohol, namely, 1,3‐diphenylprop‐2‐en‐1‐ol, were observed in all of the control experiments, suggesting that the C=C reduction of the *α*,*β*‐unsaturated ketone was more dominant than the reduction of the C=O bond.[^54^, ^55^] Notably, no desired product was obtained in the reaction of benzyl alcohol and 1‐phenylethanol in the presence of 2,2,6,6‐tetramethylpiperidin‐1‐yl)oxyl (TEMPO) as a radical scavenger (Figure 9f). Therefore, it was concluded that the anaerobic coupling of aromatic alcohols under transition‐metal‐free conditions can involve a radical mechanism, consistent with the earlier proposals on the base‐mediated conversion of alcohols.[^49^, ^56^, ^57^]

**Figure 10:**
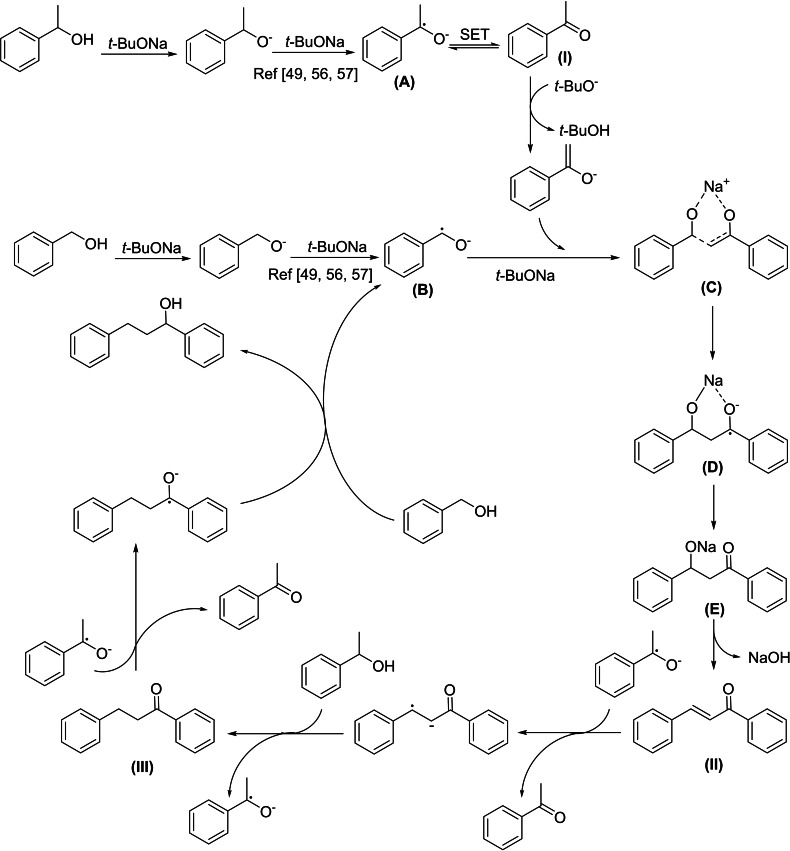
Plausible mechanism for the *β*‐alkylation of 1‐phenylethanol and benzyl alcohol in the presence of *t‐*BuONa.

Based on the obtained results and related previous literature reports, the pathway for the *β*‐alkylation of secondary alcohol with primary alcohol was proposed (Figure 10). In detail, aromatic alcohols were rapidly deprotonated to the alkoxides by *t‐*BuONa. It was suggested that these alkoxide species underwent the second deprotonation by *t‐*BuONa at the elevated temperature to generate the dianions, which subsequently convert into the radical anions (**A** and **B**) *via* a single electron transfer (SET) to the alcohol substrates or the aromatic solvent.[^49^, ^57^, ^58^] After the radical initiations, the reversible transformation of **A** to acetophenone **I** was possible due to the observation of this stable intermediate species in the reaction course. The benzyl‐based radical anion **B** can insert into the enolate form of **I**, through radical transition states **C** and **D**, to form the corresponding ketone **E** which then undergoes elimination under the basic condition, affording chalcone **II**.[^49^, ^56^] In the next steps, the *α*,*β*‐unsaturated ketone can join the further single electron transfer with the radical anions and react with the alcohol to produce the saturated ketone **III**. A similar pathway was applied to the hydrogenation of **III** to the final product and the regeneration of the radical anion **B**.

With the optimized reaction conditions in hand, the *β*‐alkylation of 1‐phenylethanol with a variety of primary alcohols was carried out to extend the scope of the method (Table 1). A good isolation yield of 1,3‐diphenylpropan‐1‐ol of 79 % was obtained from the standard reaction of 1‐phenylethanol with benzyl alcohol (**Entry 1**). Especially, the electron‐rich and electron‐deficient functional groups introduced in the *para‐*position of benzyl alcohols could well tolerate the reaction and give high yields of desired products of 80–82 % (Entries 2–4). However, *ortho*‐substituted benzyl alcohols including 2‐methylbenzyl alcohol and 2‐chlorobenzyl alcohol gave the corresponding products in slightly lower yields of 77 % and 62 %, respectively (Entries 5–6). The reason might be the chloro‐ and methyl‐ substituents being much closer to the hydroxyl group, which caused steric hindrance for the coupling reaction. Indeed, previous studies also reported that either a longer reaction time or a higher catalyst loading was required for the cross‐coupling between *ortho*‐substituted benzyl alcohols and 1‐phenylethanol due to their steric factor.[^16^, ^59^] Notably, meta‐substituted benzyl alcohols including 3‐methylbenzyl alcohol and 3‐trifluoromethylbenzyl alcohol diminished the yield of the corresponding products to 67 % and 36 % respectively (Entries 7–8). This could be explained due to the presence of high electron‐withdrawing functional groups such as 3‐trifluoromethyl suppressing the stability of benzylic intermediates through the electronic effect. The reactivity of other primary alcohols was discovered. A low tolerance for furfuryl alcohol was observed with a poor yield of 23 % (Entry 9). However, the reaction of 1‐phenylethanol with 1‐butanol yielded only a small amount of the corresponding coupling product which could not be isolated. For the case of secondary alcohol derivatives, 1‐(2‐naphthyl) ethanol showed a higher reactivity compared to 1‐phenylethanol, giving a moderate yield of 54 % for the target product (Entry 11). Unfortunately, the efforts on other secondary alcohols including 1‐(3‐nitrophenyl)ethanol and 2‐octanol (Entries 11–13) were unsuccessful probably due to the low stability of the corresponding radicals involved in the reaction. However, the effect of the functional group on the secondary alcohol reactivity and the approaches to improve their reaction should be further investigated.

**Table 1:** *β*‐alkylation of 1‐phenylethanol with primary alcohols in the presence of *t*‐BuONa.^[a]^

Entry	Reactant 1	Reactant 2	Product	Isolated yield (%)
1	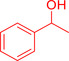	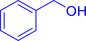	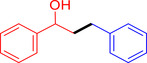	79 %
2	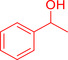	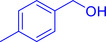	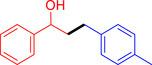	80 %
3	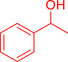	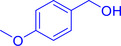	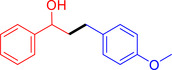	82 %
4	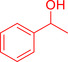	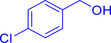	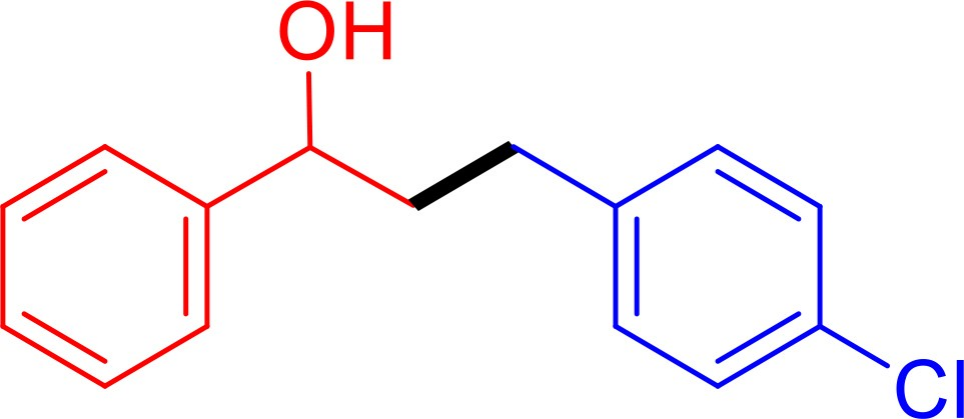	80 %
5	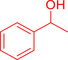	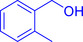	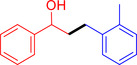	77 %
6	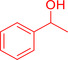	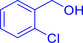	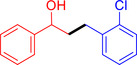	62 %
7	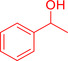	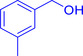	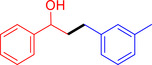	67 %
8	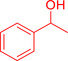	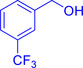	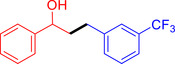	36 %
9	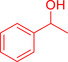	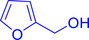	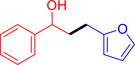	23 %
10	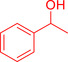	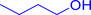	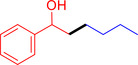	Trace
11	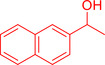	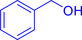	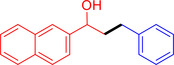	54 %
12	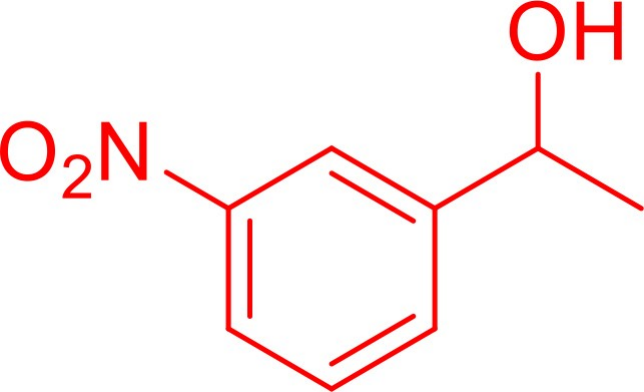	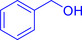	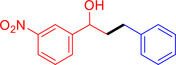	Not detected
13	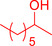	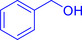	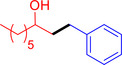	Not detected

^[a]^ Reaction conditions: 1‐arylethanol (0.75 mmol); primary alcohol (0.5 mmol); *t‐*BuONa (0.2 equiv.); toluene (0.75 mL); 140 °C; argon atmosphere, 20 h.

## Conclusions

In summary, 1,3‐diphenylpropan‐1‐ol was successfully synthesized through the *β*‐alkylation of secondary alcohols with primary alcohols under basic conditions with the absence of a transition metal catalyst. The control experiments proposed that *t*‐BuONa could be used to deprotonate both the primary and secondary alcohols, followed by the formation and subsequent addition of radical anions to obtain *β*‐alkylated alcohols. Notably, this radical coupling annulation can enable the use of readily available and less toxic alcohols as alkylating agents for the selective C−C bond formation and prevent the environmental impact and contamination of transition metal species. Furthermore, this work demonstrates a new, green, and efficient methodology with corresponding 1,3‐diphenylpropan‐1‐ol derivatives in isolated yields of 23–82 %. Further expanding the substrate scope in terms of primary aliphatic alcohols and secondary alcohols as well as upscaling the reaction have been currently under investigation.

## Supporting Information

Synthetic procedure, characterization of compounds.

## Conflict of Interests

The authors declare no conflict of interest.

1

## Supporting information

As a service to our authors and readers, this journal provides supporting information supplied by the authors. Such materials are peer reviewed and may be re‐organized for online delivery, but are not copy‐edited or typeset. Technical support issues arising from supporting information (other than missing files) should be addressed to the authors.

Supporting Information

## Data Availability

The data that support the findings of this study are available in the supplementary material of this article.
