# Supplementary material for: Green Synthesis of Diphenyl‐Substituted Alcohols Via Radical Coupling of Aromatic Alcohols Under Transition‐Metal‐Free Conditions
Source: ChemistryOpen. 2024 Aug 22;13(12):e202400139. doi: 10.1002/open.202400139 (PMC11625937; doi:10.1002/open.202400139)
Supplement: Supplementary file 1 — Supporting Information [file OPEN-13-e202400139-s001.pdf]

ChemistryOpen

Supporting Information

Green Synthesis of Diphenyl-Substituted Alcohols *Via* Radical Coupling of Aromatic Alcohols Under Transition-Metal-Free Conditions

Ha V. Le, Vy T. B. Nguyen, Huy X. Le, Tung T. Nguyen, Khoa D. Nguyen, Phuoc H. Ho,* and Thuong T. H. Nguyen*

Supporting Information

Green Synthesis of Diphenyl-Substituted Alcohols *Via* Radical Coupling of Aromatic Alcohols Under Transition-Metal-Free Conditions

Ha V. Le,[a, b] Vy T. B. Nguyen,[a, b] Huy X. Le,[a, b] Tung T. Nguyen,[a, b] Khoa D. Nguyen,[a, b] Phuoc H. Ho,*[c] and Thuong T. H. Nguyen,* [a, b]

[a] Faculty of Chemical Engineering, Ho Chi Minh City University of Technology (HCMUT), 268 Ly Thuong Kiet, District 10, Ho Chi Minh City, Vietnam

[b] Vietnam National University Ho Chi Minh City, Linh Trung Ward, Thu Duc City, Ho Chi Minh City, Vietnam

[c] Chemical Engineering, Competence Centre for Catalysis, Chalmers University of Technology, Gothenburg, SE-412 96, Sweden

1,3-diphenylpropan-1-ol

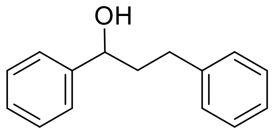

Prepared as the described procedure using 1-phenylethanol (91.6 mg, 0.75 mmol), benzyl alcohol (54.1 mg, 0.5 mmol), sodium *tert*-butoxide (9.6 mg, 0.1 equiv.) at 140 ℃ under an argon atmosphere for 20 h. TLC silica gel 60 F_{254}, R_f = 0.30 (ethyl acetate:hexane = 1:5 (v./v.). Purified by column chromatography on silica gel (230-400 mesh or 37-63 µm, ethyl acetate/hexane = 1:5 (v./v.), yielding an oily liquid (83.8 mg, yield = 79%).

^{1}H NMR (600 MHz, $CDCl_3$) δ (ppm) 7.37-7.22 (m, 7H), 7.20-7.15 (m, 3H), 4.67 (dd, *J* = 7.9, 5.3 Hz, 1H), 2.77-2.72 (m, 1H), 2.68-2.63 (m, 1H), 2.15-2.09 (m, 1H), 2.05-1.50 (m, 2H).

^{13}C NMR (126 MHz, $CDCl_3$) δ (ppm) 144.60, 141.81, 128.54, 128.47, 128.42, 127.67, 125.95, 125.88, 73.91, 40.48, 32.08.

1-phenyl-3-(p-tolyl)propan-1-ol

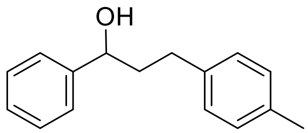

Prepared as the described procedure using 1-phenylethanol (91.6 mg, 0.75 mmol), 4-methylbenzyl alcohol (61.1 mg, 0.5 mmol), sodium *tert*-butoxide (9.6 mg, 0.1 equiv.) at 140 ℃ under an argon atmosphere for 20 h. TLC silica gel 60 F_{254}, R_f = 0.32 (ethyl acetate:hexane = 1:5 (v./v.). Purified by column chromatography on silica gel (230-400 mesh or 37-63 µm, ethyl acetate/hexane = 1:5 (v./v.), yielding an oily liquid (90.5 mg, yield = 80%).

^{1}H NMR (600 MHz, $CDCl_3$) δ (ppm) 7.38-7.22 (m, 5H), 7.08 (s, 4H), 4.67 (dd, *J* = 7.8, 5.3 Hz, 1H), 2.77-2.66 (m, 1H), 2.65-2.60 (m, 1H), 2.31 (s, 3H), 2.16-2.07 (m, 1H), 2.04-1.98 (m, 1H), 1.77 (s, 1H).

^{13}C NMR (126 MHz, $CDCl_3$) δ (ppm) 144.64, 138.68, 135.31, 129.10, 128.52, 128.33, 127.63, 125.96, 73.93, 40.58, 31.62, 21.00.

3-(4-methoxyphenyl)-1-phenylpropan-1-ol

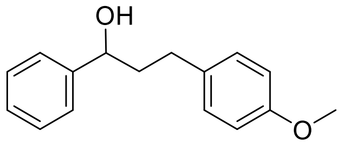

Prepared as the described procedure using 1-phenylethanol (91.6 mg, 0.75 mmol), 4-methoxybenzyl alcohol (69.1 mg, 0.5 mmol), sodium *tert*-butoxide (9.6 mg, 0.1 equiv.) at 140 ℃ under an argon atmosphere for 20 h. TLC silica gel 60 F_{254}, R_f = 0.27 (ethyl acetate:hexane = 1:5 (v./v.). Purified by column chromatography on silica gel (230-400 mesh or 37-63 µm, ethyl acetate/hexane = 1:5 (v./v.), yielding an oily liquid (99.3 mg, yield = 82%).

^{1}H NMR (600 MHz, $CDCl_3$) δ (ppm) 7.37-7.23 (m, 5H), 7.15-7.05 (m, 2H), 6.86-6.78 (m, 2H), 4.66 (dd, *J* = 7.9, 5.3 Hz, 1H), 3.77 (s, 3H), 2.74-2.55 (m, 2H), 2.13 – 2.04 (m, 1H), 2.03 – 1.74 (m, 3H).

^{13}C NMR (126 MHz, $CDCl_3$) δ (ppm) 144.64, 138.68, 135.31, 129.10, 128.52, 128.33, 127.63, 125.96, 73.93, 40.58, 31.62, 21.00.

3-(4-chlorophenyl)-1-phenylpropan-1-ol

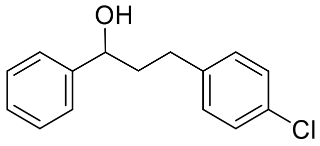

Prepared as the described procedure using 1-phenylethanol (91.6 mg, 0.75 mmol), 4-chlorobenzyl alcohol (71.3 mg, 0.5 mmol), sodium *tert*-butoxide (9.6 mg, 0.1 equiv.) at 140 ℃ under an argon atmosphere for 20 h. TLC silica gel 60 F_{254}, R_f = 0.25 (ethyl acetate:hexane = 1:8 (v./v.). Purified by column chromatography on silica gel (230-400 mesh or 37-63 µm, ethyl acetate/hexane = 1:8 (v./v.), yielding an oily liquid (98.4 mg, yield = 80%).

^{1}H NMR (600 MHz, $CDCl_3$) δ (ppm) 7.34-7.29 (m, 4H), 7.28-7.18 (m, 3H), 7.08 (d, *J* = 8.3 Hz, 2H), 4.66-4.56 (m, 1H), 2.70-2.65 (m, 1H), 2.64-2.56 (m, 1H), 2.11-1.63 (m, 3H).

^{13}C NMR (126 MHz, $CDCl_3$) δ (ppm) 144.47, 140.30, 131.57, 129.83, 128.58, 128.49, 127.73, 125.94, 73.68, 40.32, 31.39.

1-phenyl-3-(o-tolyl)propan-1-ol

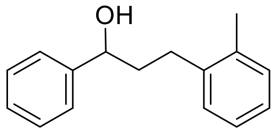

Prepared as the described procedure using 1-phenylethanol (91.6 mg, 0.75 mmol), 2-methylbenzyl alcohol (61.1 mg, 0.5 mmol), sodium *tert*-butoxide (9.6 mg, 0.1 equiv.) at 140 ℃ under an argon atmosphere for 20 h. TLC silica gel 60 F_{254}, R_f = 0.24 (ethyl acetate:hexane = 1:10 (v./v.). Purified by column chromatography on silica gel (230-400 mesh or 37-63 µm, ethyl acetate/hexane = 1:10 (v./v.), yielding an oily liquid (87.1 mg, yield = 77%).

^{1}H NMR (600 MHz, $CDCl_3$) δ (ppm) 7.34-7.30 (m, 4H), 7.27-7.23 (m, 1H), 7.13-7.04 (m, 4H), 4.66 (dd, J = 7.5, 5.6 Hz, 1H), 2.74-2.69 (m, 1H), 2.61-2.55 (m, 1H), 2.23 (s, 3H), 2.15-1.89 (m, 3H).

^{13}C NMR (126 MHz, $CDCl_3$) δ (ppm) 144.67, 140.09, 136.02, 130.28, 128.82, 128.57, 127.70, 126.01, 74.23, 39.30, 29.47, 19.28.

3-(2-chlorophenyl)-1-phenylpropan-1-ol

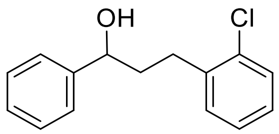

Prepared as the described procedure using 1-phenylethanol (91.6 mg, 0.75 mmol), 2-chlorobenzyl alcohol (71.3 mg, 0.5 mmol), sodium *tert*-butoxide (9.6 mg, 0.1 equiv.) at 140 ℃ under an argon atmosphere for 20 h. TLC silica gel 60 F_{254}, R_f = 0.21 (ethyl acetate:hexane = 1:10 (v./v.). Purified by column chromatography on silica gel (230-400 mesh or 37-63 µm, ethyl acetate/hexane = 1:10 (v./v.), yielding an oily liquid (76.3 mg, yield = 62%).

^{1}H NMR (600 MHz, $CDCl_3$) δ (ppm) 7.39-7.25 (m, 6H), 7.24-7.20 (m, 1H), 7.18-7.15 (m, 1H), 7.14-7.11 (m, 1H), 4.72 (dd, J = 7.9, 5.2 Hz, 1H), 2.92-2.87 (m, 1H), 2.80-2.75 (m, 1H), 2.23-1.78 (m, 3H).

^{13}C NMR (126 MHz, $CDCl_3$) δ (ppm) 144.42, 139.45, 133.97, 130.42, 129.52, 128.54, 127.68, 127.39, 126.79, 125.91, 74.00, 38.74, 30.05.

1-phenyl-3-(m-tolyl)propan-1-ol

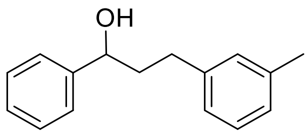

Prepared as the described procedure using 1-phenylethanol (91.6 mg, 0.75 mmol), 3-methylbenzyl alcohol (61.1 mg, 0.5 mmol), sodium *tert*-butoxide (9.6 mg, 0.1 equiv.) at 140 ℃ under an argon atmosphere for 20 h. TLC silica gel 60 F_{254}, R_f = 0.25 (ethyl acetate:hexane = 1:10 (v./v.). Purified by column chromatography on silica gel (230-400

mesh or 37-63 µm, ethyl acetate/hexane = 1:10 (v./v.), yielding an oily liquid (75.8 mg, yield = 67%).

^{1}H NMR (600 MHz, $CDCl_3$) δ (ppm) 7.40-7.29 (m, 4H), 7.31-7.23 (m, 1H), 7.16 (t, *J* = 7.5 Hz, 1H), 6.99 (dd, *J* = 8.1, 5.3 Hz, 3H), 4.74-4.62 (m, 1H), 2.73-2.69 (m, 1H), 2.66-2.58 (m, 1H), 2.31 (s, 3H), 2.15-2.09 (m, 1H), 2.07-1.98 (m, 1H), 1.88 (s, 1H).

^{13}C NMR (126 MHz, $CDCl_3$) δ (ppm) 144.63, 141.73, 137.95, 129.27, 128.52, 128.30, 127.63, 126.61, 125.95, 125.44, 73.97, 40.52, 32.01, 21.40.

1-phenyl-3-(3-(trifluoromethyl)phenyl)propan-1-ol

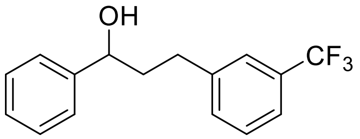

Prepared as the described procedure using 1-phenylethanol (91.6 mg, 0.75 mmol), (3-(trifluoromethyl)phenyl)methanol (88.1 mg, 0.5 mmol), sodium *tert*-butoxide (9.6 mg, 0.1 equiv.) at 140 ℃ under an argon atmosphere for 20 h. TLC silica gel 60 F_{254}, R_f = 0.25 (ethyl acetate:hexane = 1:10 (v./v.). Purified by column chromatography on silica gel (230-400 mesh or 37-63 µm, ethyl acetate/hexane = 1:10 (v./v.), yielding an oily liquid (50.4 mg, yield = 36%).

^{1}H NMR (600 MHz, $CDCl_3$) δ (ppm) 7.48-7.40 (m, 2H), 7.40-7.26 (m, 7H), 4.69 (dd, *J* = 7.9, 5.2 Hz, 1H), 2.92-2.67 (m, 2H), 2.21-1.99 (m, 2H), 1.90 (s, 1H).

^{13}C NMR (151 MHz, $CDCl_3$) δ (ppm) 144.31, 142.73, 131.84, 128.77, 128.62, 127.83, 125.87, 125.15, 125.12, 122.78, 122.75, 73.76, 40.20, 31.86.

1-(naphthalen-2-yl)-3-phenylpropan-1-ol

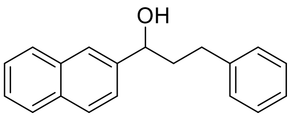

Prepared as the described procedure using 1-(naphthalen-2-yl)ethan-1-ol (128.3 mg, 0.75 mmol), benzyl alcohol (54.1 mg, 0.5 mmol), sodium *tert*-butoxide (9.6 mg, 0.1 equiv.) at 140 ℃ under an argon atmosphere for 20 h. TLC silica gel 60 F_{254}, R_f = 0.3 (ethyl acetate:hexane = 1:10 (v./v.). Purified by column chromatography on silica gel (230-400

mesh or 37-63 µm, ethyl acetate/hexane = 1:10 (v./v.), yielding a white solid (71.1 mg, yield = 54%).

^{1}H NMR (600 MHz, $CDCl_3$) δ (ppm) 7.87-7.78 (m, 3H), 7.76 (d, *J* = 1.7 Hz, 1H), 7.51-7.42 (m, 3H), 7.30-7.24 (m, 2H), 7.21-7.15 (m, 3H), 4.83 (dd, *J* = 7.7, 5.4 Hz, 1H), 2.81-2.64 (m, 2H), 2.28-2.07 (m, 2H), 2.03 (s, 1H).

^{13}C NMR (151 MHz, $CDCl_3$) δ (ppm) 141.94, 141.79, 133.34, 133.08, 128.49, 128.44, 128.41, 127.97, 127.73, 126.21, 125.91, 125.90, 124.71, 124.09, 74.01, 40.35, 32.09.

3-(furan-2-yl)-1-phenylpropan-1-ol

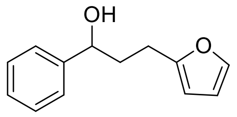

Prepared as the described procedure using 1-phenylethanol (91.6 mg, 0.75 mmol), furan-2-ylmethanol (49.1 mg, 0.5 mmol), sodium *tert*-butoxide (9.6 mg, 0.1 equiv.) at 140 ℃ under an argon atmosphere for 20 h. TLC silica gel 60 F_{254}, R_f = 0.2 (ethyl acetate:hexane = 1:10 (v./v.). Purified by column chromatography on silica gel (230-400 mesh or 37-63 µm, ethyl acetate/hexane = 1:10 (v./v.), yielding an oily liquid (23.3 mg, yield = 23%).

^{1}H NMR (600 MHz, $CDCl_3$) δ (ppm) 7.39-7.32 (m, 4H), 7.32-7.26 (m, 2H), 6.27 (dd, *J* = 3.2, 1.8 Hz, 1H), 6.00 (d, *J* = 3.0 Hz, 1H), 4.70 (dd, *J* = 7.9, 5.3 Hz, 1H), 2.85-2.58 (m, 2H), 2.26 -1.91 (m, 2H).

^{13}C NMR (126 MHz, $CDCl_3$) δ (ppm) 155.55, 144.35, 140.96, 128.54, 127.69, 125.88, 110.13, 105.03, 73.71, 37.18, 24.41.

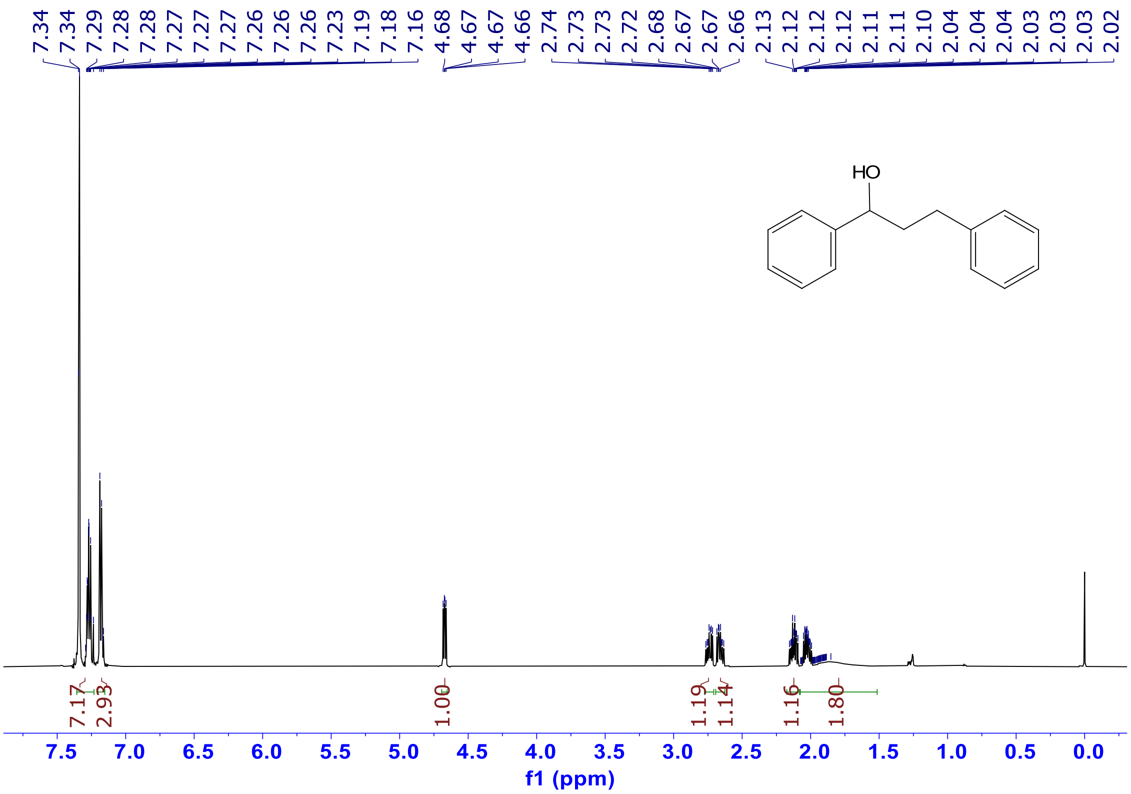

Figure S1. ^{1}H-NMR spectrum of 1,3-diphenylpropan-1-ol.

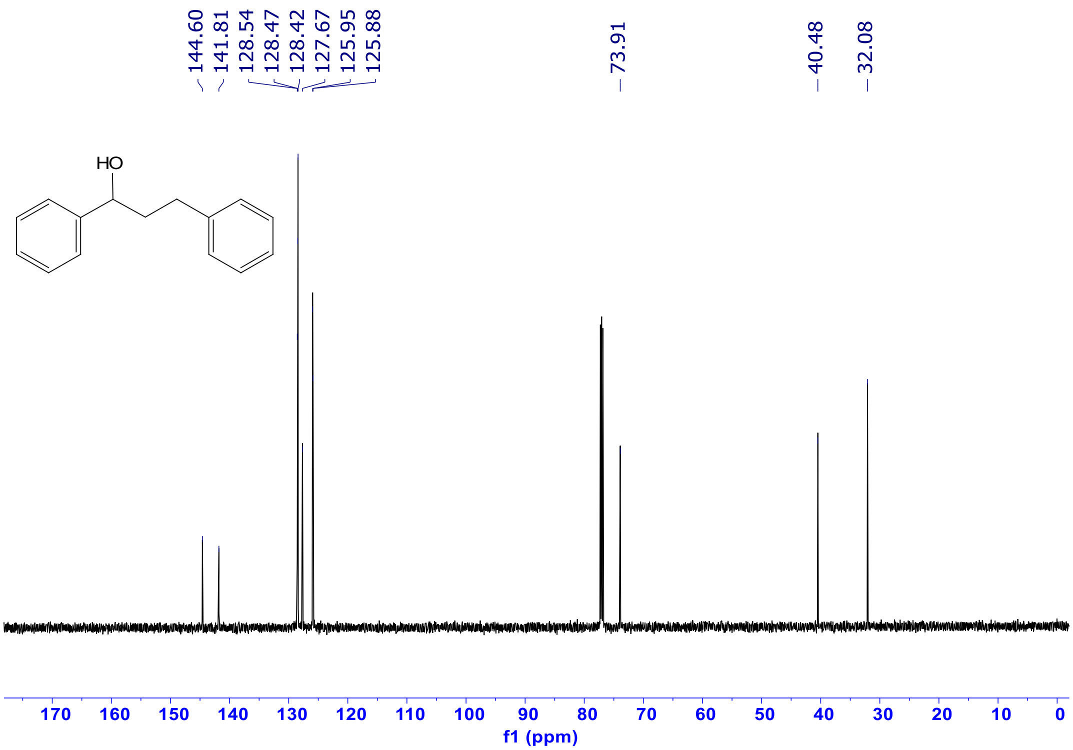

Figure S2. ^{13}C-NMR spectrum of 1,3-diphenylpropan-1-ol.

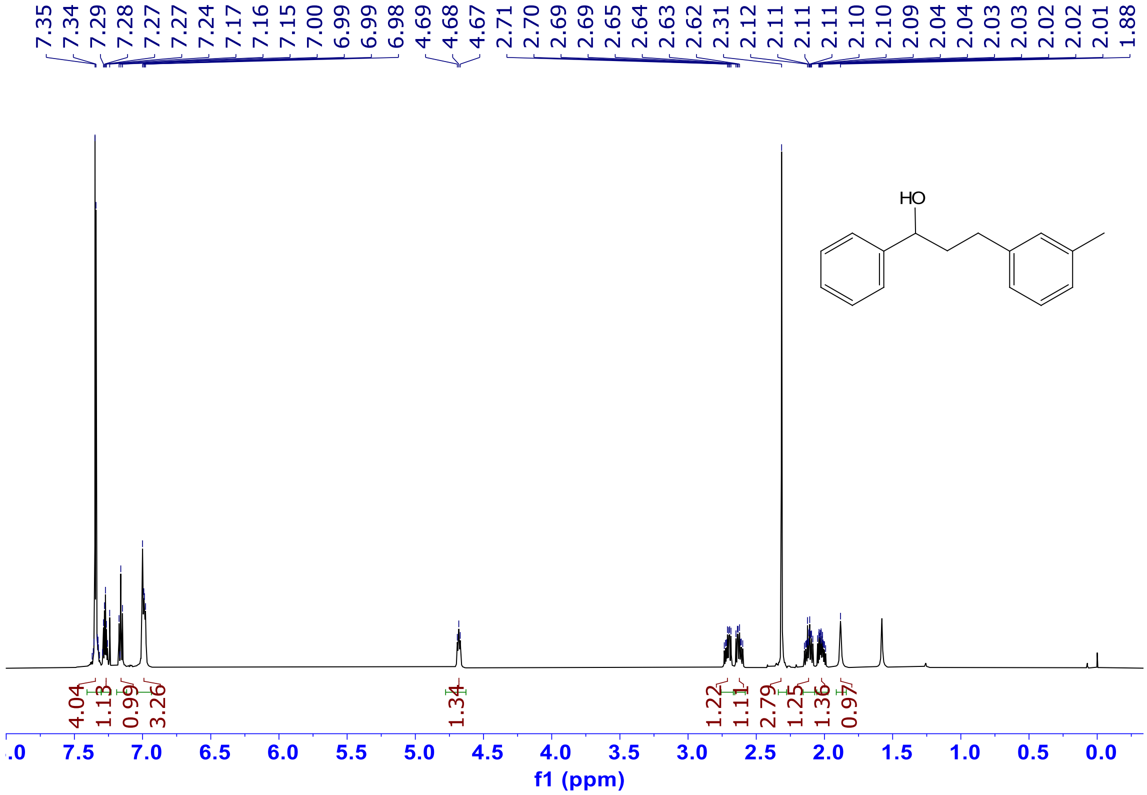

Figure S3. ^{1}H-NMR spectrum of 1-phenyl-3-(m-tolyl)propan-1-ol.

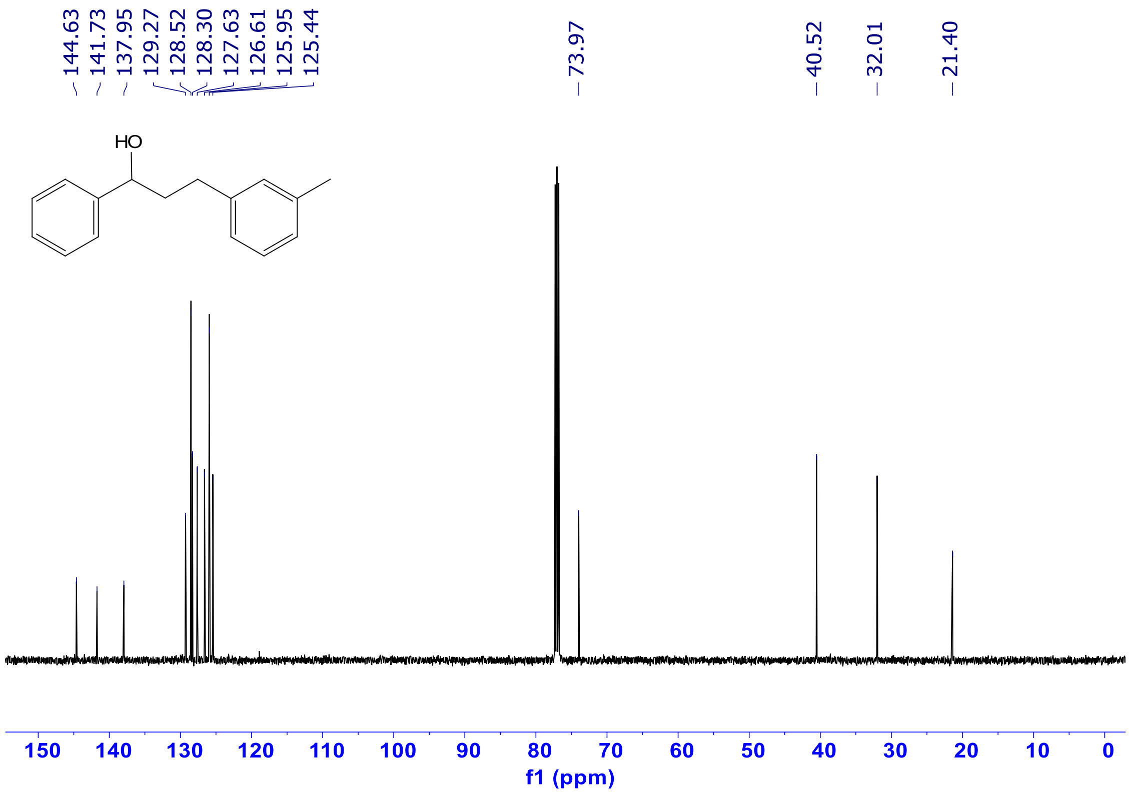

Figure S4. ^{13}C-NMR spectrum of 1-phenyl-3-(m-tolyl)propan-1-ol.

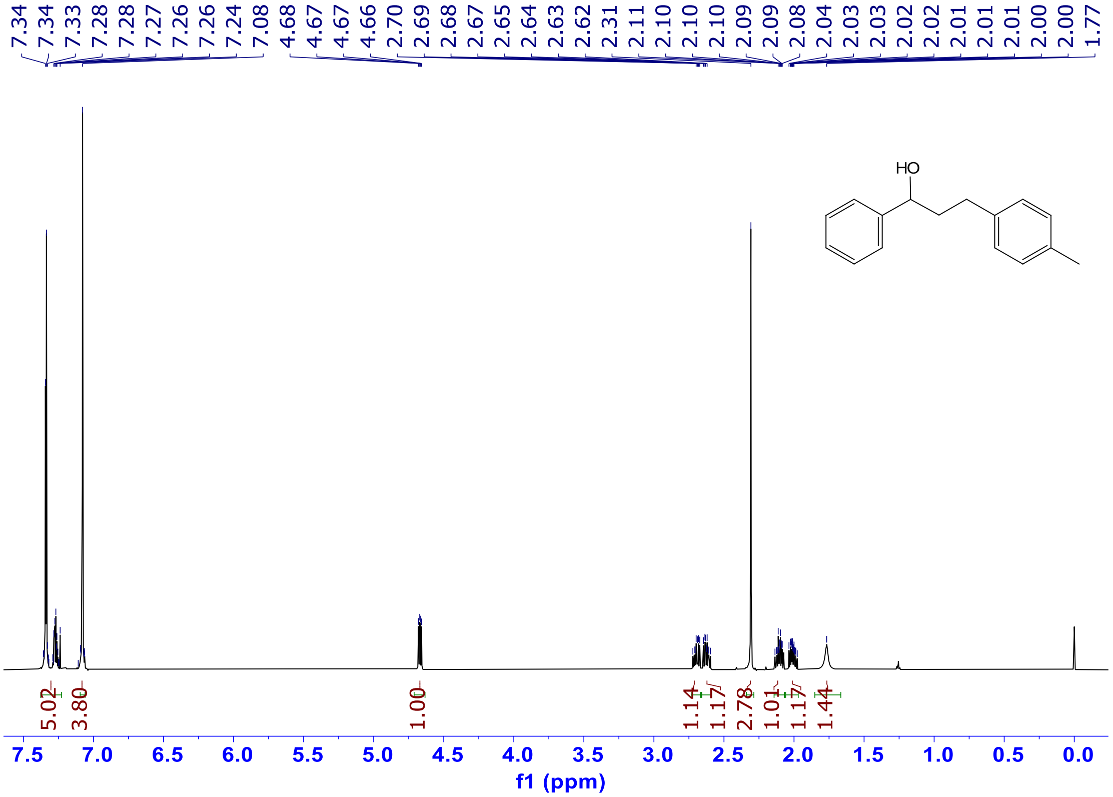

Figure S5. ^{1}H-NMR spectrum of 1-phenyl-3-(p-tolyl)propan-1-ol.

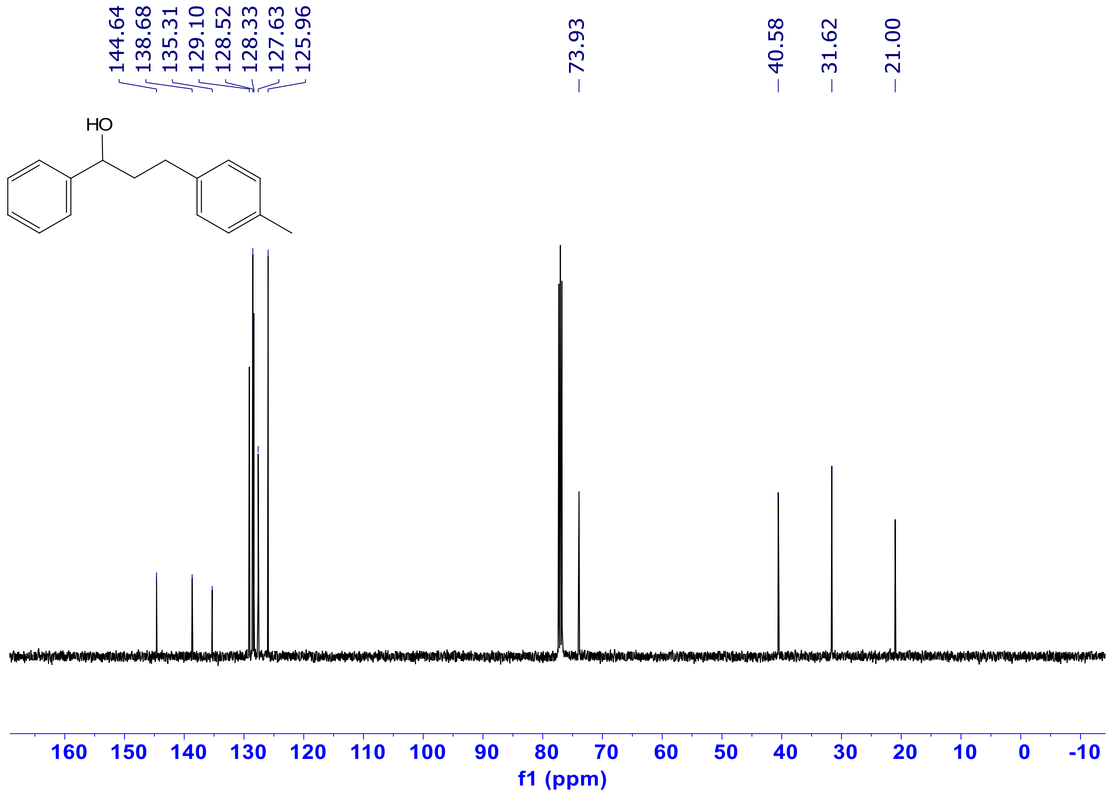

Figure S6. ^{13}C-NMR spectrum of 1-phenyl-3-(p-tolyl)propan-1-ol.

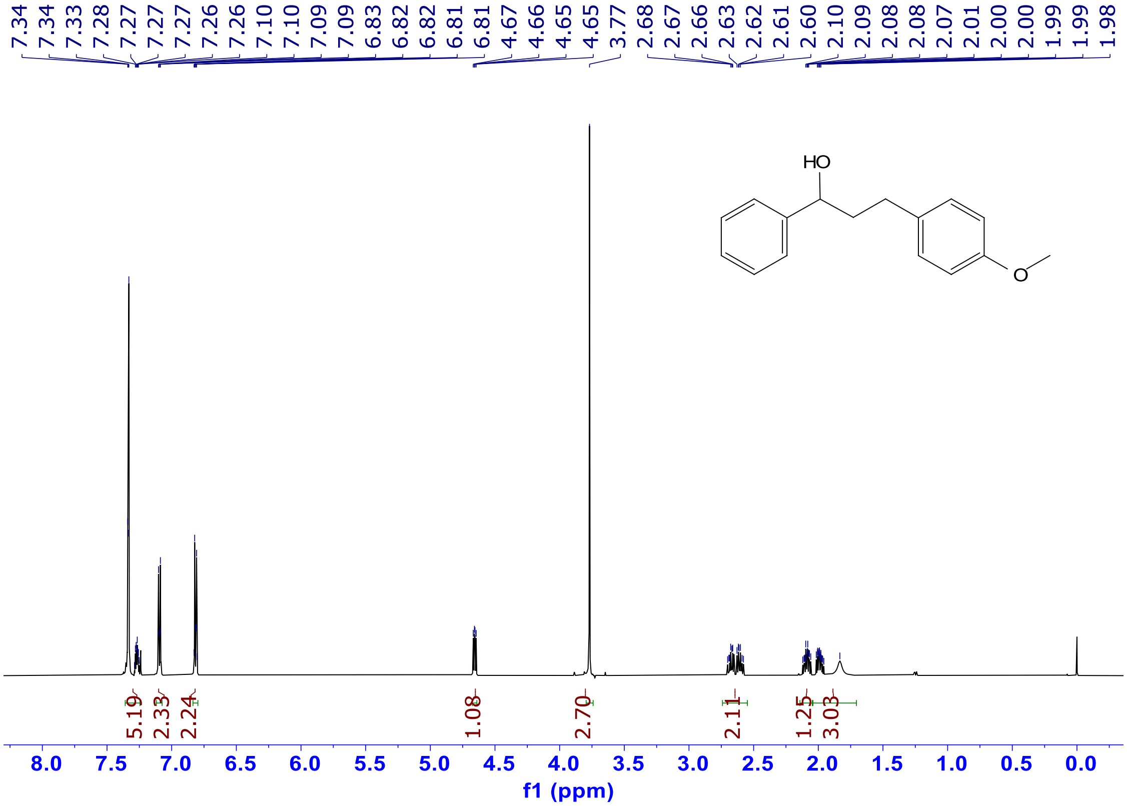

Figure S7. ^{1}H-NMR spectrum of 3-(4-methoxyphenyl)-1-phenylpropan-1-ol.

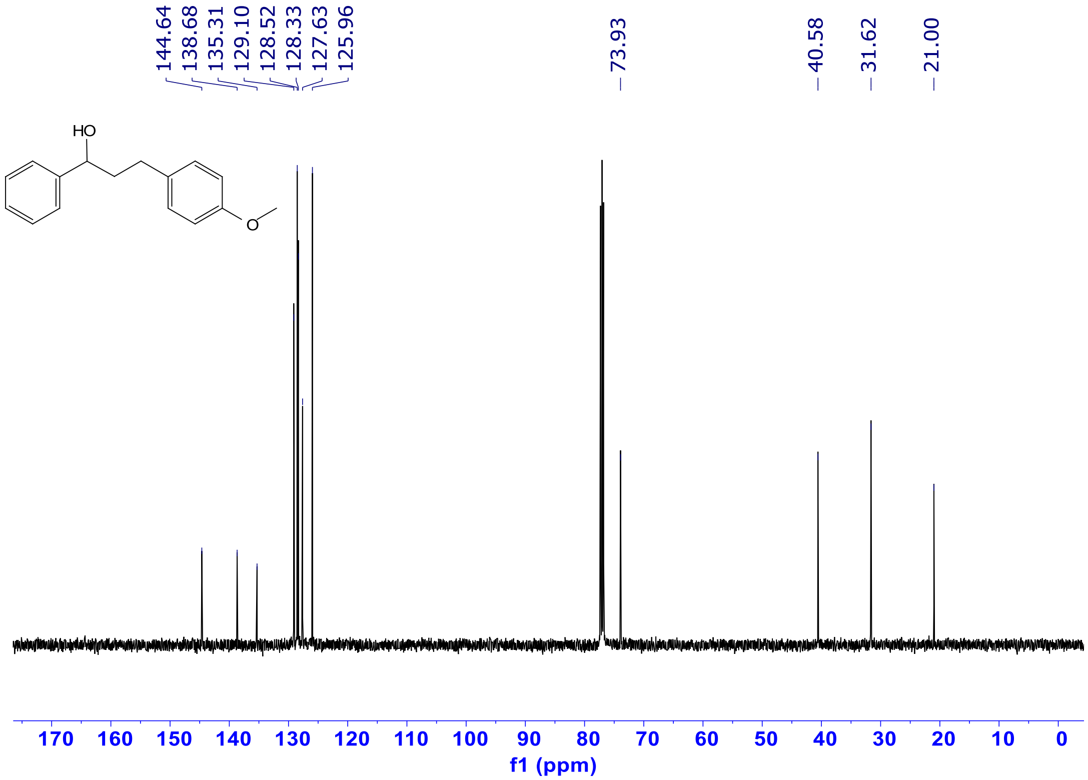

Figure S8. ^{13}C-NMR spectrum of 3-(4-methoxyphenyl)-1-phenylpropan-1-ol.

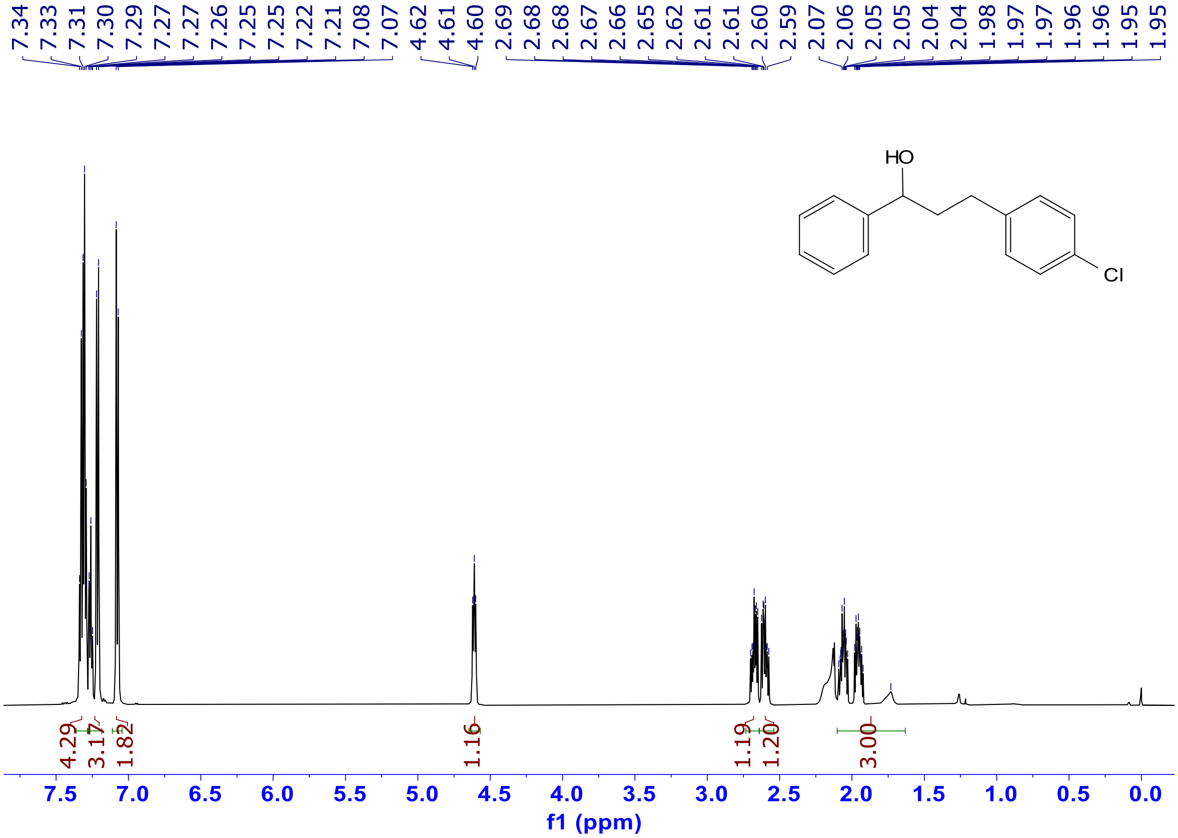

Figure S9. ^{1}H-NMR spectrum of 3-(4-chlorophenyl)-1-phenylpropan-1-ol.

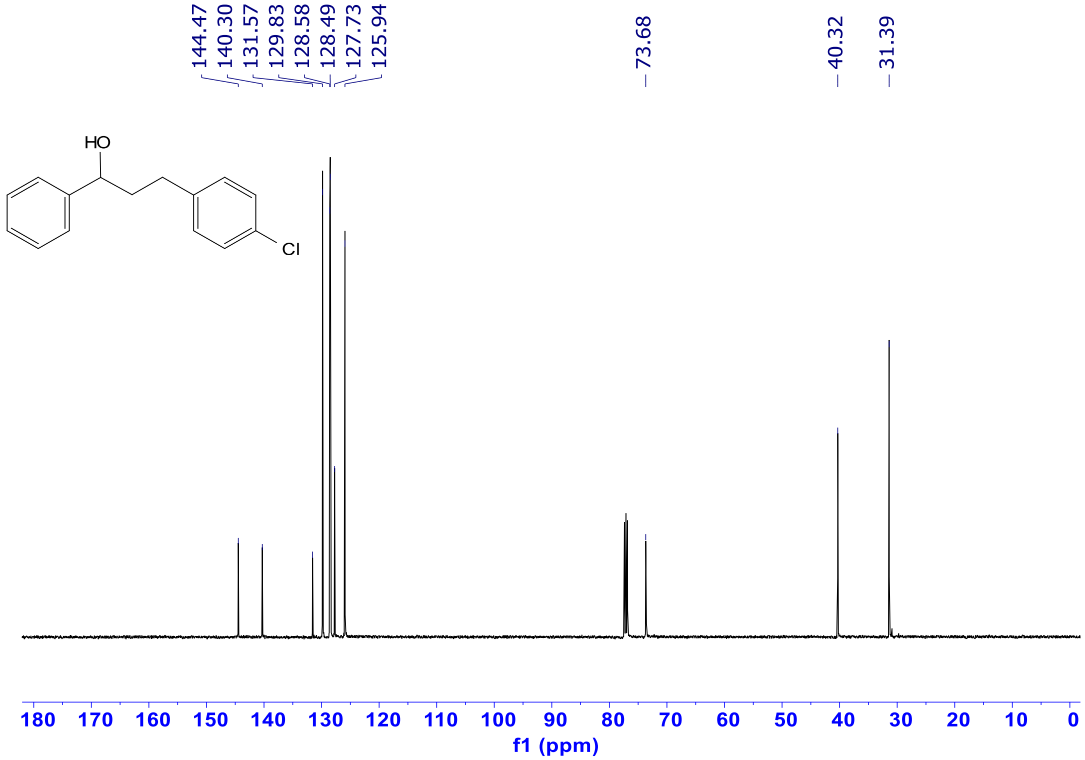

Figure S10. ^{13}C-NMR spectrum of 3-(4-chlorophenyl)-1-phenylpropan-1-ol.

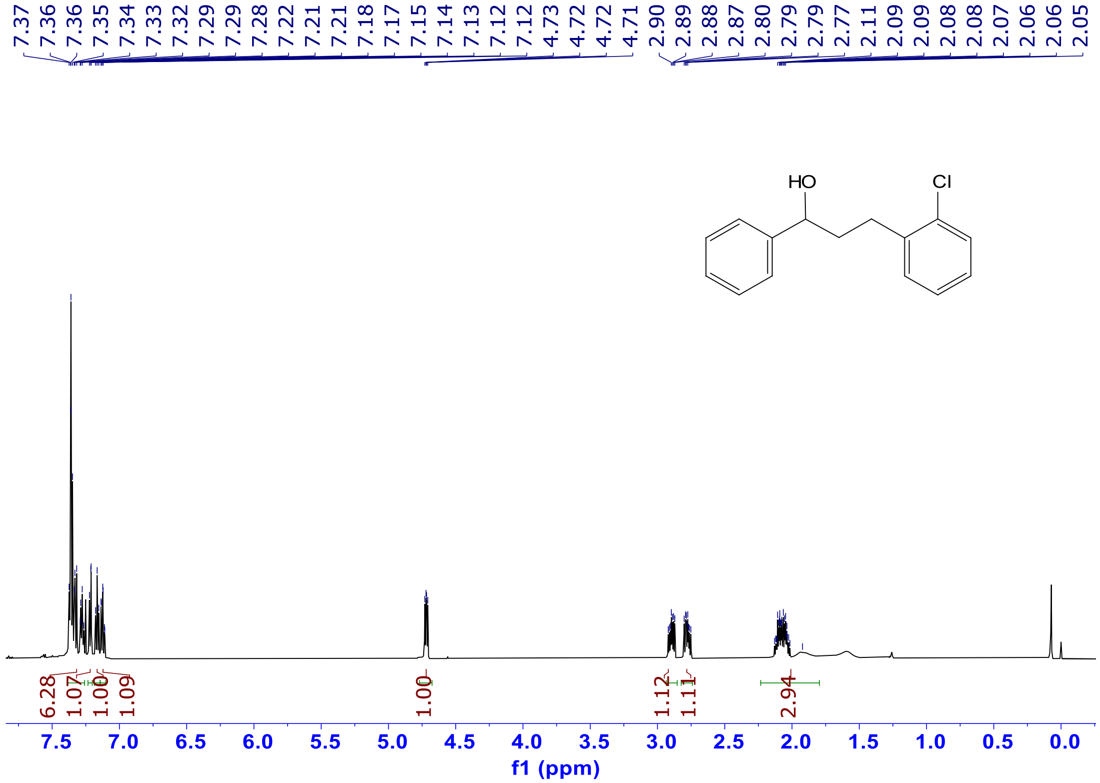

Figure S11. ^{1}H-NMR spectrum of 3-(2-chlorophenyl)-1-phenylpropan-1-ol.

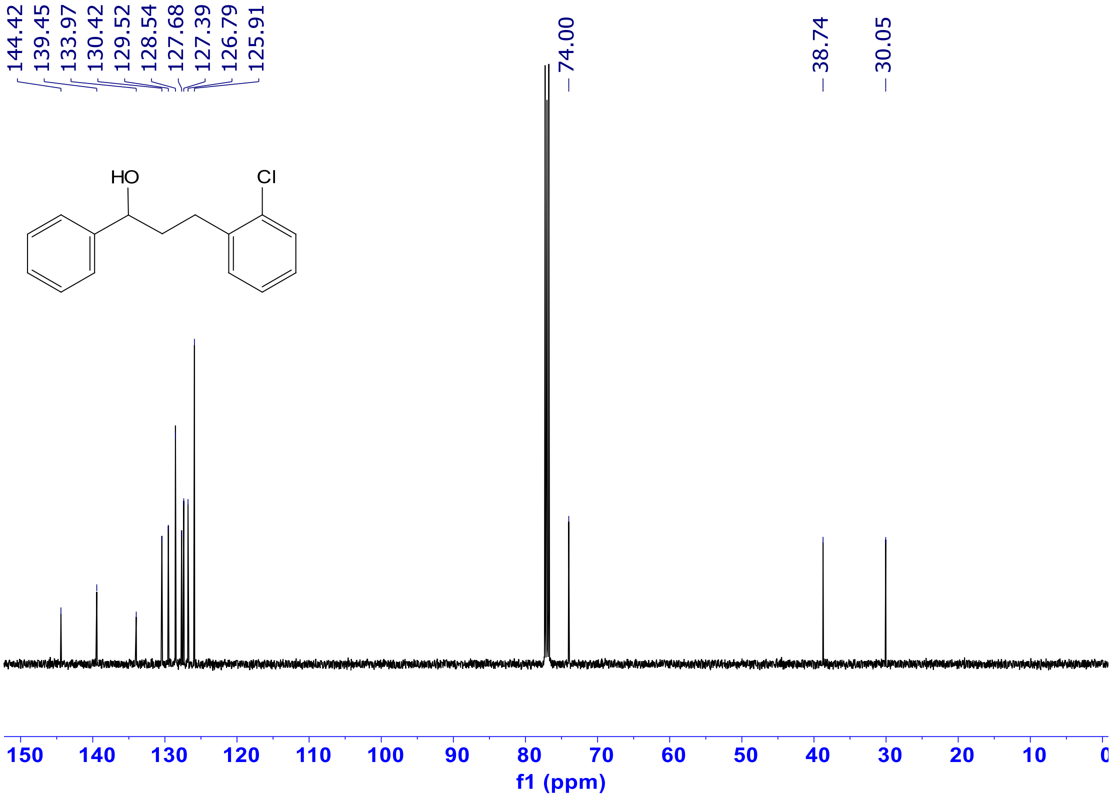

Figure S12. ^{13}C-NMR spectrum of 3-(2-chlorophenyl)-1-phenylpropan-1-ol.

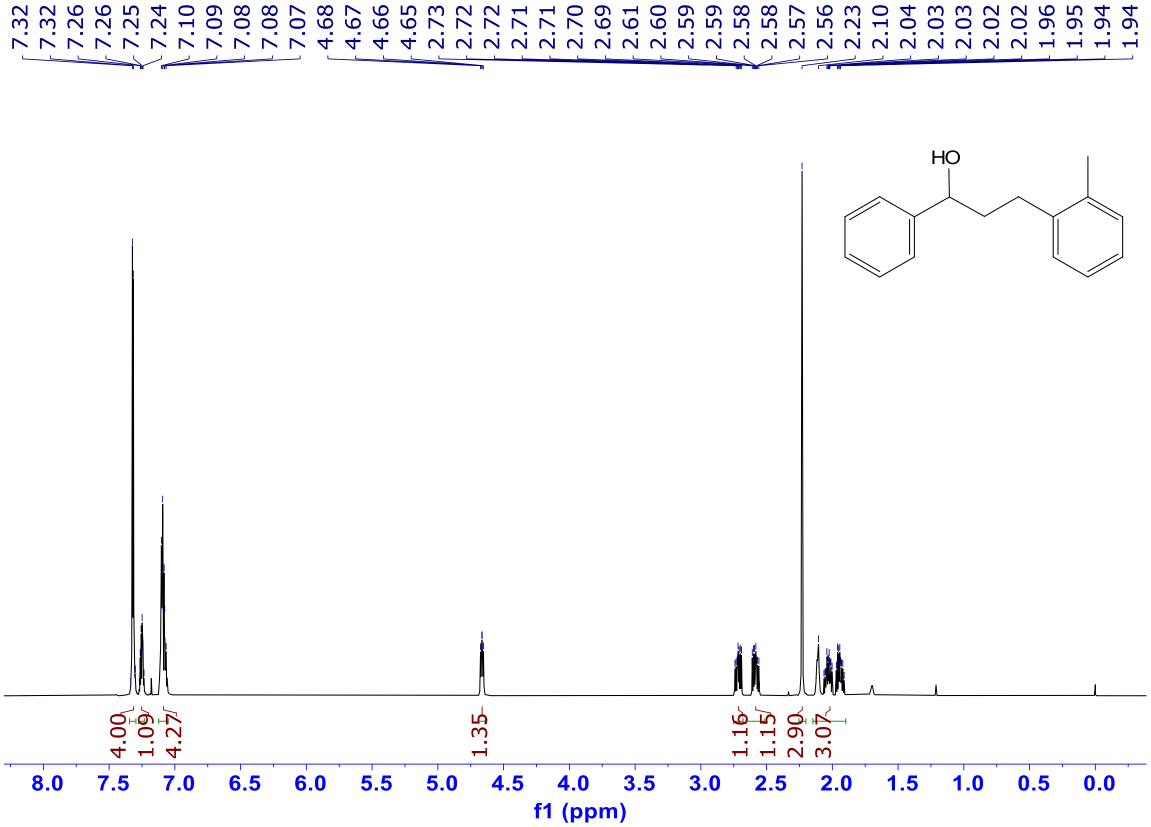

Figure S13. ^{1}H-NMR spectrum of 1-phenyl-3-(o-tolyl)propan-1-ol.

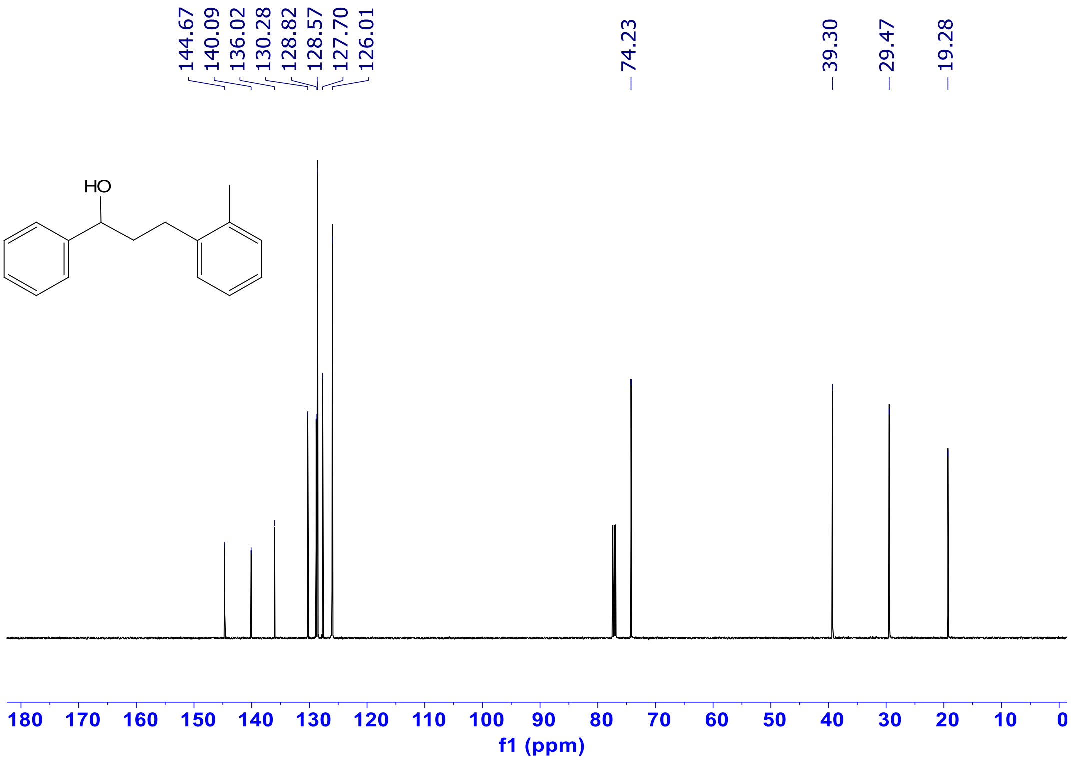

Figure S14. ^{13}C-NMR spectrum of 1-phenyl-3-(o-tolyl)propan-1-ol.

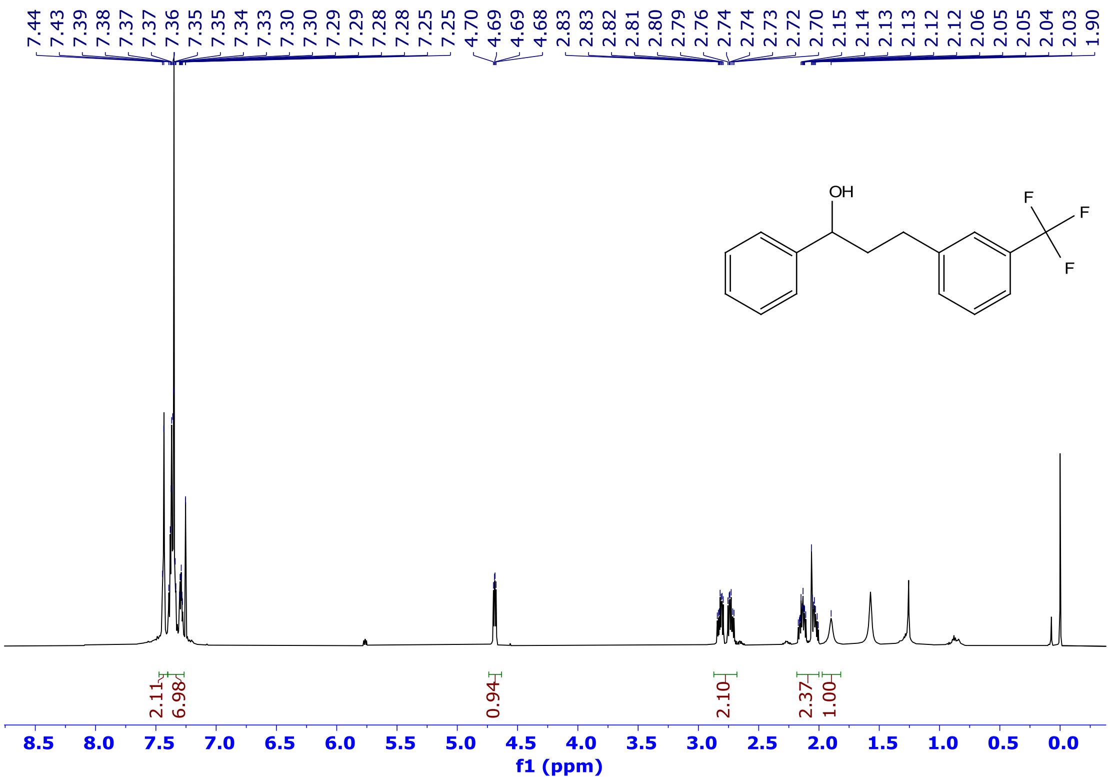

Figure S15. ^{1}H-NMR spectrum of 1-phenyl-3-(3-(trifluoromethyl)phenyl)propan-1-ol.

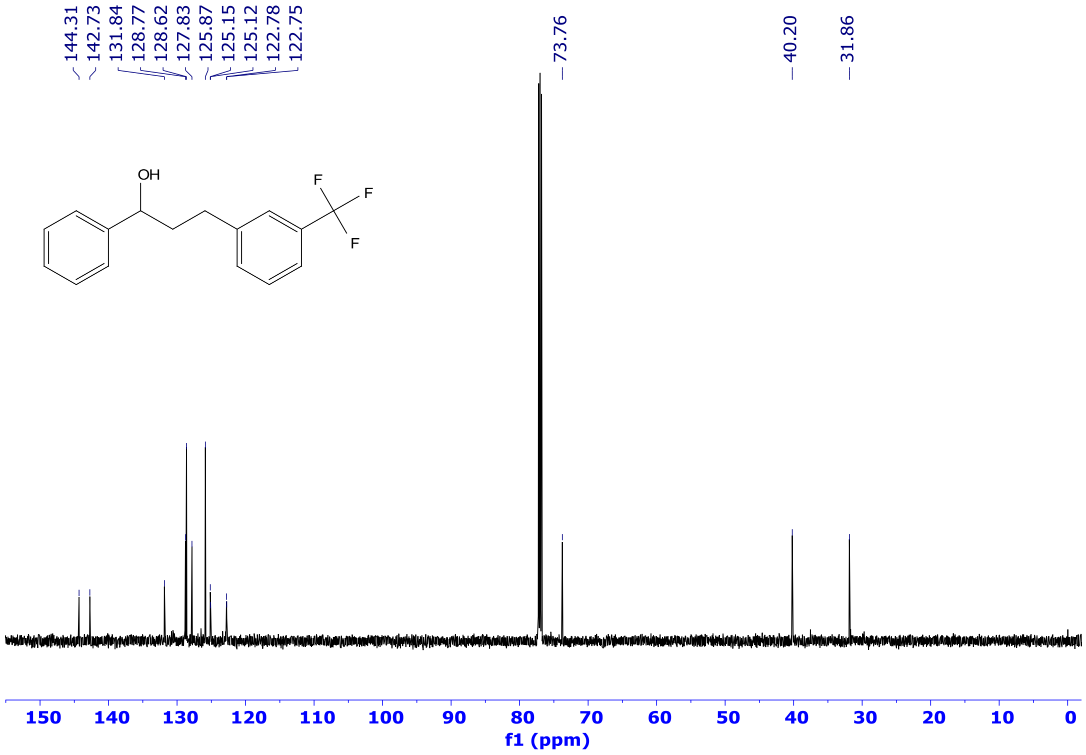

Figure S16. ^{13}C-NMR spectrum of 1-phenyl-3-(3-(trifluoromethyl)phenyl)propan-1-ol.

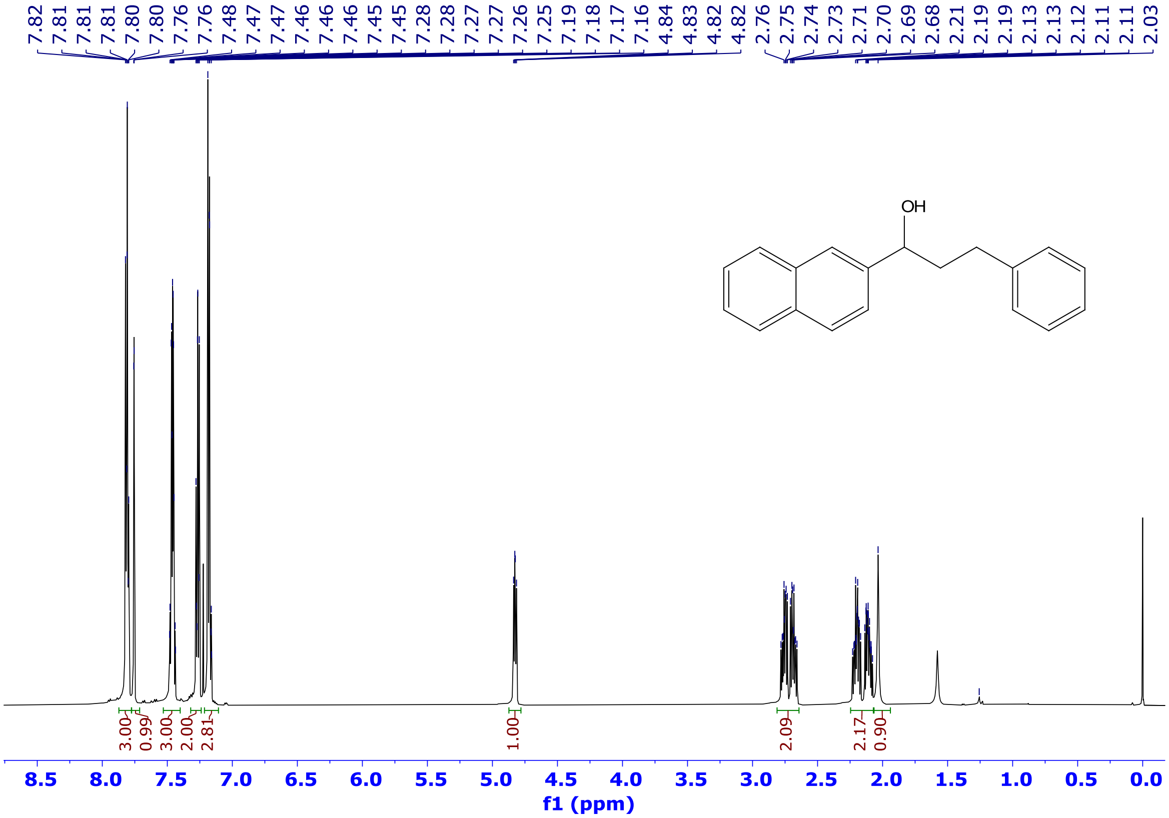

Figure S17. ^{1}H-NMR spectrum of 1-(naphthalen-2-yl)-3-phenylpropan-1-ol.

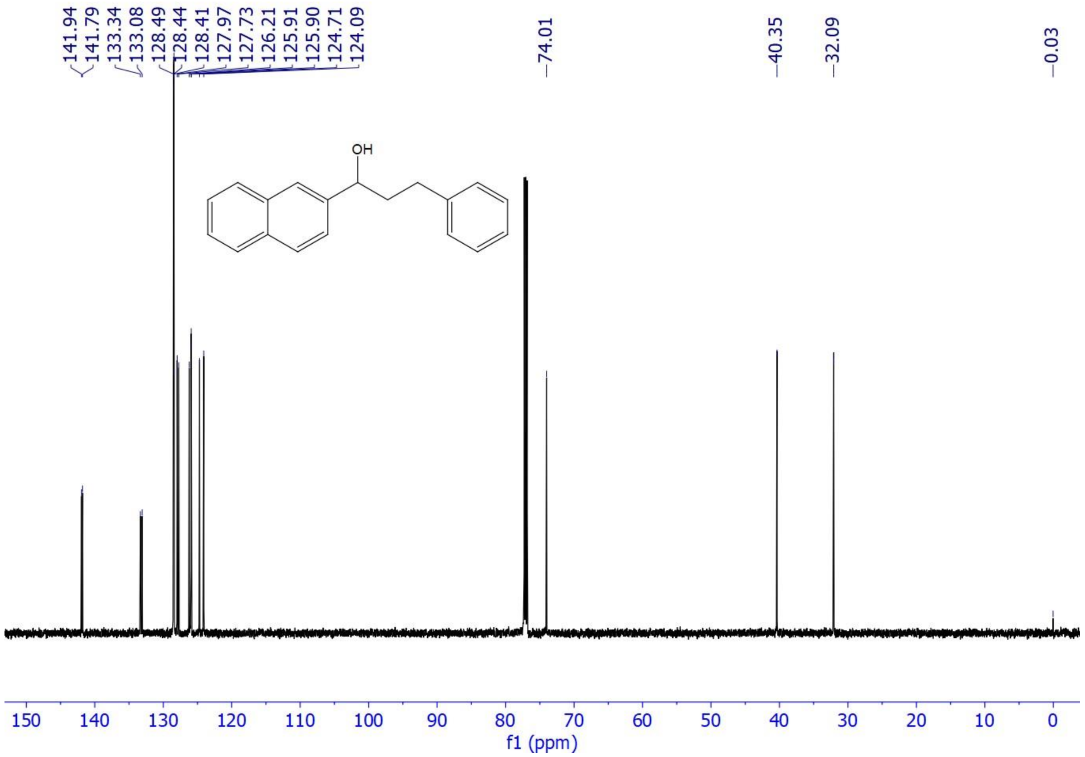

Figure S18. ^{13}C-NMR spectrum of 1-(naphthalen-2-yl)-3-phenylpropan-1-ol.

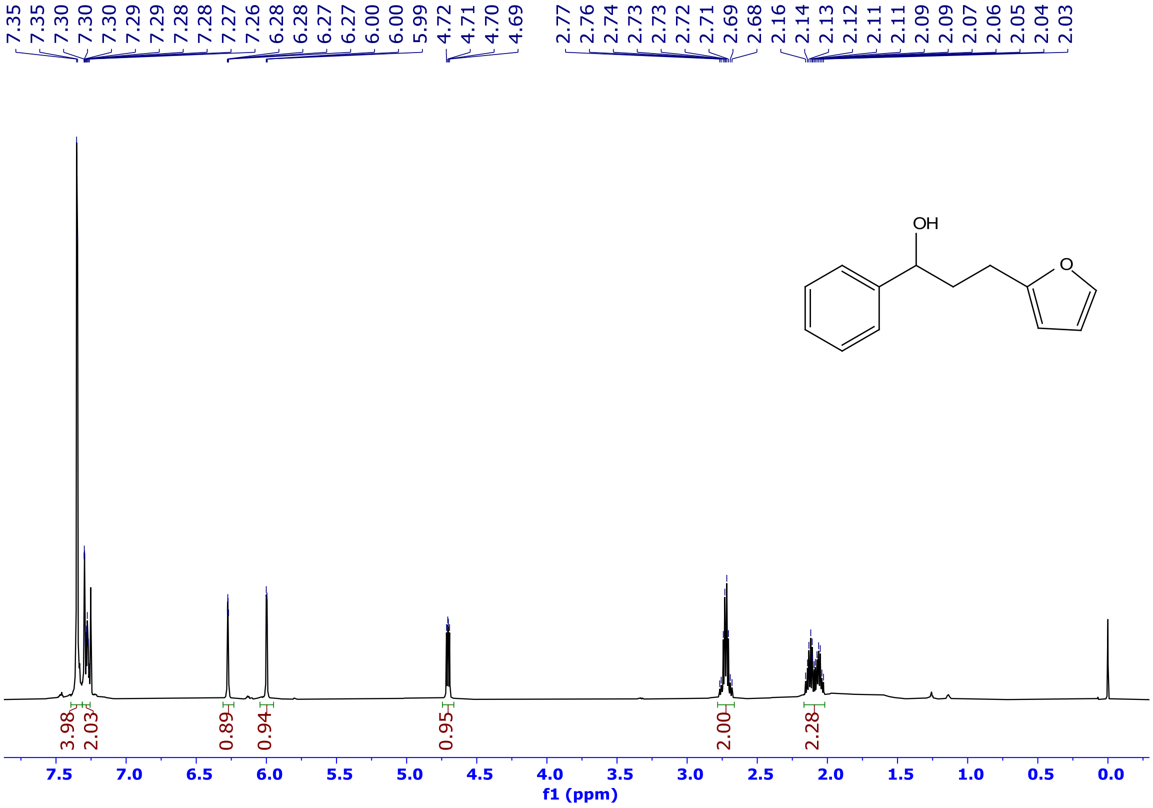

Figure S19. ^{1}H-NMR spectrum of 3-(furan-2-yl)-1-phenylpropan-1-ol.

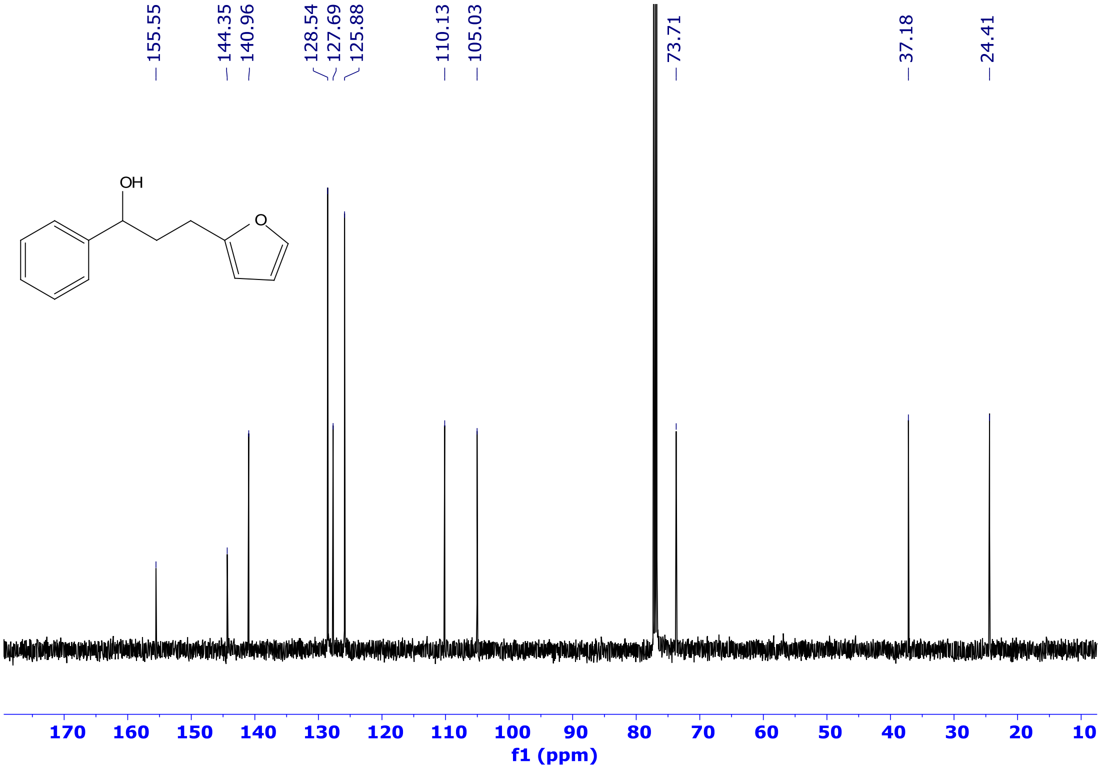

Figure S20. ^{13}C-NMR spectrum of 3-(furan-2-yl)-1-phenylpropan-1-ol.
